# A comprehensive influenza reporter virus panel for high-throughput deep profiling of neutralizing antibodies

**DOI:** 10.1038/s41467-021-21954-2

**Published:** 2021-03-19

**Authors:** Adrian Creanga, Rebecca A. Gillespie, Brian E. Fisher, Sarah F. Andrews, Julia Lederhofer, Christina Yap, Liam Hatch, Tyler Stephens, Yaroslav Tsybovsky, Michelle C. Crank, Julie E. Ledgerwood, Adrian B. McDermott, John R. Mascola, Barney S. Graham, Masaru Kanekiyo

**Affiliations:** 1 Vaccine Research Center, National Institute of Allergy and Infectious Diseases, National Institutes of Health, Bethesda, MD USA; 2 Electron Microscopy Laboratory, Cancer Research Technology Program, Frederick National Laboratory for Cancer Research sponsored by the National Cancer Institute, Frederick, MD USA

**Keywords:** Immunological techniques, Microbiology techniques, Influenza virus

## Abstract

Broadly neutralizing antibodies (bnAbs) have been developed as potential countermeasures for seasonal and pandemic influenza. Deep characterization of these bnAbs and polyclonal sera provides pivotal understanding for influenza immunity and informs effective vaccine design. However, conventional virus neutralization assays require high-containment laboratories and are difficult to standardize and roboticize. Here, we build a panel of engineered influenza viruses carrying a reporter gene to replace an essential viral gene, and develop an assay using the panel for in-depth profiling of neutralizing antibodies. Replication of these viruses is restricted to cells expressing the missing viral gene, allowing it to be manipulated in a biosafety level 2 environment. We generate the neutralization profile of 24 bnAbs using a 55-virus panel encompassing the near-complete diversity of human H1N1 and H3N2, as well as pandemic subtype viruses. Our system offers in-depth profiling of influenza immunity, including the antibodies against the hemagglutinin stem, a major target of universal influenza vaccines.

## Abstract (web summary)

Understanding the human antibody response to influenza A virus strains is important for vaccine development. Here, Creanga et al. generate a panel of 55 replication-deficient reporter viruses representing diversity of human H1N1 and H3N2, and pandemic subtypes and characterize the neutralization profile of 24 antibodies and polyclonal sera.

## Introduction

Influenza virus continues to cause seasonal epidemics and pandemics despite vaccine availability. In addition, highly pathogenic avian influenza viruses cause sporadic outbreaks in humans with a high mortality rate, posing potential risk of human adaptation and are considered pandemic threats^1^. Viral glycoprotein hemagglutinin (HA) is required for viral entry into the host cell through binding to sialic acid moieties on glycoproteins or glycolipids and mediates fusion of the viral membrane with host endosomal membranes. Another viral glycoprotein neuraminidase (NA) cleaves sialic acid and promotes the release of progeny viruses from infected cells. Neutralizing antibodies are primarily directed against HA and can compete for receptor binding, inhibit the membrane fusion machinery, or cause virus aggregation. Antibodies to NA can sometimes have neutralizing activity but are thought to primarily limit virus egress by inhibiting enzymatic activity and release of virus thereby inhibiting viral spread. Based on the genetic and antigenic properties of HA and NA, influenza A viruses are divided into groups 1 and 2, each of which have several subtypes defined primarily by HA. To date, there are 18 HA and 11 NA subtypes identified and characterized^2^. Currently, strains of H1N1 and H3N2 influenza A as well as influenza B viruses co-circulate in humans. In addition, several other subtypes of animal influenza viruses (e.g., H5N1, H6N1, H7N9, H9N2, and H10N8) can also infect humans and occasionally result in mortality. In contrast to influenza A viruses, which have an extensive zoonotic reservoir, influenza B viruses are isolated almost exclusively from humans with a more limited evolutionary history and have diverged into only two genetically and antigenically distinct lineages (Victoria- and Yamagata-like lineages)^3^.

The discovery of broadly neutralizing antibodies (bnAbs) capable of neutralizing multiple influenza virus subtypes in humans opens an opportunity for developing a universal influenza vaccine, which elicits such antibodies^4–6^. Many of these antibodies target conserved epitopes in the HA stem and neutralize virus by inhibiting the viral fusion machinery, therefore, the activity is not detectable by traditional hemagglutination inhibition (HAI) assay, which measures the ability of antibody to inhibit virus–receptor interaction. Several less broad bnAbs recognize the receptor-binding site (RBS) in the HA head, hence exhibiting HAI activity^7^. Since most universal influenza vaccine candidates aim to induce protective levels of such bnAb response^6,8^, comprehensive analysis of the neutralization breadth and potency regardless of HAI activity is crucial to accelerate the efforts to develop effective universal influenza vaccines. The influenza microneutralization (MN) assay has been the most commonly used method to measure neutralizing activity of antibodies against influenza virus^9^. In this assay, virus replication is measured by either detecting viral nucleoprotein (NP) with an enzyme-linked immunosorbent assay (ELISA), titrating hemagglutination, or scoring cytopathic effects. Plaque-reduction neutralization assay is also commonly used for influenza and other viruses. These approaches are labor-intensive, not easily scalable, and the handling of live viruses of animal origin requires high-containment laboratories. There is also significant performance variability between laboratories due to multi-step signal amplification or reliance on subjective scoring. Given these inevitable limitations of the current MN assay, there is a need to transform the MN assay to be safe, high throughput, robust, easy to standardize, and automation compatible^10^.

The use of reporter viruses for developing standardized high-throughput neutralization assays has greatly advanced our ability to measure and characterize antibody responses induced by infection and/or vaccination^11–13^. While HA/NA pseudotyped reporter lentiviruses have been constructed for influenza and utilized effectively for measuring neutralizing activity^14^, they are often criticized for not recapitulating some of the key aspects of influenza viruses, such as viral morphology and HA spike density^15,16^. Therefore, building influenza viruses with a reporter feature is an attractive alternative. Among several approaches to produce influenza reporter viruses, a replication-competent reporter virus can be developed by fusing a reporter gene to a viral gene (e.g., PA or NS)^17^. This new virus remains as virulent as the parental virus in animals, making it suitable for viral pathogenesis studies, yet still subject to biosafety precautions. In contrast, single-cycle infectious or replication-restricted reporter (R3) influenza viruses can be generated by replacing one of the viral essential genes (e.g., PB1 or HA) with a reporter gene^17–20^. Thereby these R3 viruses are capable of replicating only in cells complementing the deleted viral gene product in trans, making it safe to handle in low-containment laboratories.

In the present study, we developed a comprehensive panel of R3 viruses to enable high-throughput and in-depth influenza virus neutralization profiling. We generated a neutralization matrix of 24 monoclonal antibodies (mAbs) and 55 R3 viruses spanning 6 subtypes of influenza virus and compared the R3 virus-based assay with the gold-standard ELISA-based MN assay^9^. The reporter virus assay provides a more robust method for probing the breadth of anti-influenza immunity needed for developing universal influenza vaccines.

## Results

### Generation of R3∆PB1 viruses

The influenza virus has a segmented genome, which allows its rapid and relatively easy genetic manipulation. Each segment consists of either one or two protein-coding sequences flanked by short noncoding regions (NCRs). The genome packaging signal sequences are located at both 3′- and 5′-termini of each segment encompassing the entire NCRs and part of the adjacent protein-coding sequence. Genome packaging sequences are unique to each segment and required for an efficient incorporation of each viral RNA molecule into the virion. The PB1 segment encodes two proteins: PB1, the main component of the viral RNA-dependent RNA polymerase, and PB1-F2, translated from the +2 frame and not required for virus replication^21^. The protein-coding sequence of PB1 (A/WSN/1933, H1N1) has 2,274 nucleotides and is flanked by 24 bases at the 3′- and 43 bases at 5′-termini. In addition to the NCRs, genome packaging signals of the PB1 segment comprise 120 bases of the coding region at both 3′- and 5′-termini^18,22^ (Supplementary Fig. 1).

**Fig. 1:**
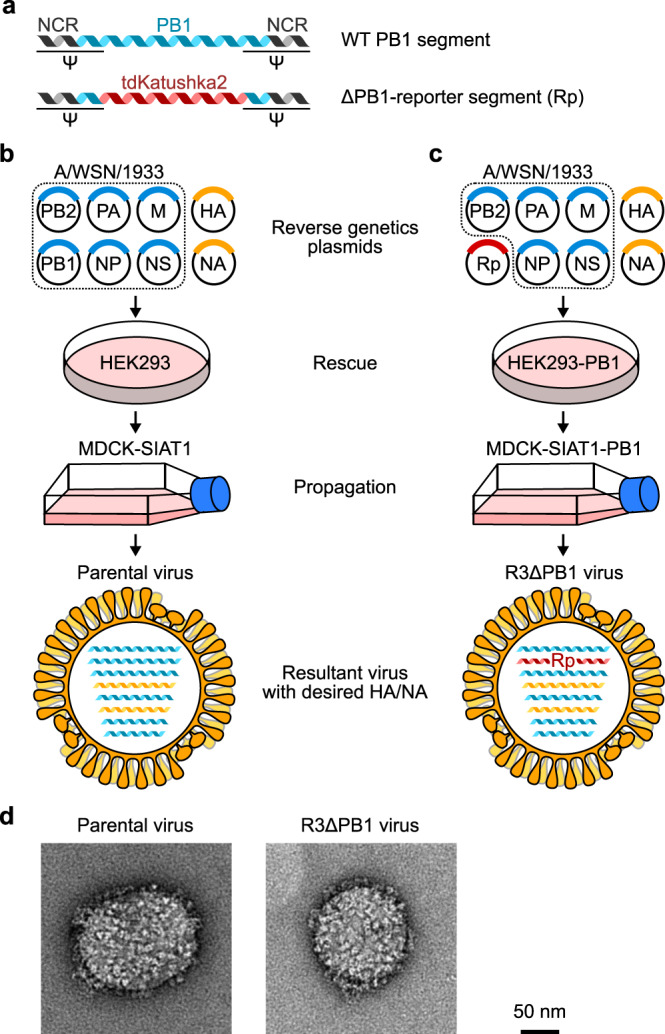
Development of replication-restricted reporter (R3) ∆PB1 influenza A virus. **a** Design of engineered PB1 segment encoding fluorescence reporter tdKatushka2. **b**, **c** Rescue of parental wild-type molecular clone (**b**) and R3∆PB1 (**c**) viruses using reverse genetics of eight plasmid system with HA and NA segments of virus of interest and internal gene segments of A/WSN/1933. PB1 segment of A/WSN/1933 is used to rescue molecularly cloned parental viruses, while engineered PB1 segment is used for R3∆PB1 influenza viruses. R3∆PB1 influenza viruses require cells expressing PB1 in trans for rescue and propagation. Rp, tdKatushka2 reporter gene-encoded segment. **d** Negative stain electron microscopy of parental molecular clone (left) and R3∆PB1 (right) of A/Michigan/45/2015 (H1N1) virus. Electron microscopic experiment was performed once and representative image of each virus is shown.

To build R3 influenza viruses, we altered the PB1 segment by removing the PB1 coding sequence not required for genome packaging and replacing it with the reporter encoding fluorescent protein tdKatushka2^23^. To prevent translation of alternative transcripts from PB1 segments, we mutated potential initiation codons (ATGs) found between the 3′ end and the reporter open reading frame (ORF) (Fig. 1a). PB1 is essential for virus replication therefore, R3 influenza viruses in which PB1 was removed (R3∆PB1) can be propagated only in cells expressing the PB1 in trans (Fig. 1b, c). Thus, we used MDCK-SIAT1 cells, which constitutively express human β-galactoside α2,6-sialyltransferase 1 (SIAT1)^24^ to prepare a cell line constitutively expressing PB1 of A/WSN/1933 by transfecting a plasmid encoding a puromycin-resistance gene, PB1, and a self-cleaving peptide derived from *Thosea asigna* virus 2A (T2A) in between the two genes. The R3∆PB1 virus was rescued by reverse genetics using 8 plasmids encoding HA and NA of wild-type H1N1 or H3N2 influenza A viruses; PB2, PA, NP, M, and NS of A/WSN/1933; and another encoding the reporter. To validate the use of R3∆PB1 viruses for influenza neutralization assays, we also rescued replication-competent H1N1 and H3N2 parental viruses, which possess HA and NA of wild-type viruses and the internal genes including PB1 of A/WSN/1933, and propagated in MDCK-SIAT1 cells (Fig. 1b, c).

To examine the morphology of R3∆PB1 virus (A/Michigan/45/2015, H1N1), we performed negative stain electron microscopy and found that there was no visible difference in size and spike density between R3∆PB1 and the corresponding wild-type molecularly cloned parental viruses, indicating that the R3∆PB1 virus remains morphologically indistinguishable from its parental virus (Fig. 1d). Virus growth kinetics of the R3∆PB1 (A/Michigan/45/2015) and its parental viruses in MDCK SIAT1 and PB1-expressing MDCK-SIAT1 cells confirmed that the R3∆PB1 virus replicates only in PB1-expressing cells, whereas parental virus replicates similarly in MDCK-SIAT1 cells with or without PB1 expression (Supplementary Fig. 2). These results demonstrate that the R3∆PB1 viruses are replication-incompetent in cells lacking PB1 expression but they replicate comparably in PB1-expressing cells to corresponding parental viruses carrying PB1 with identical HA and NA.

### Influenza virus neutralization assay using R3 virus

**Fig. 2:**
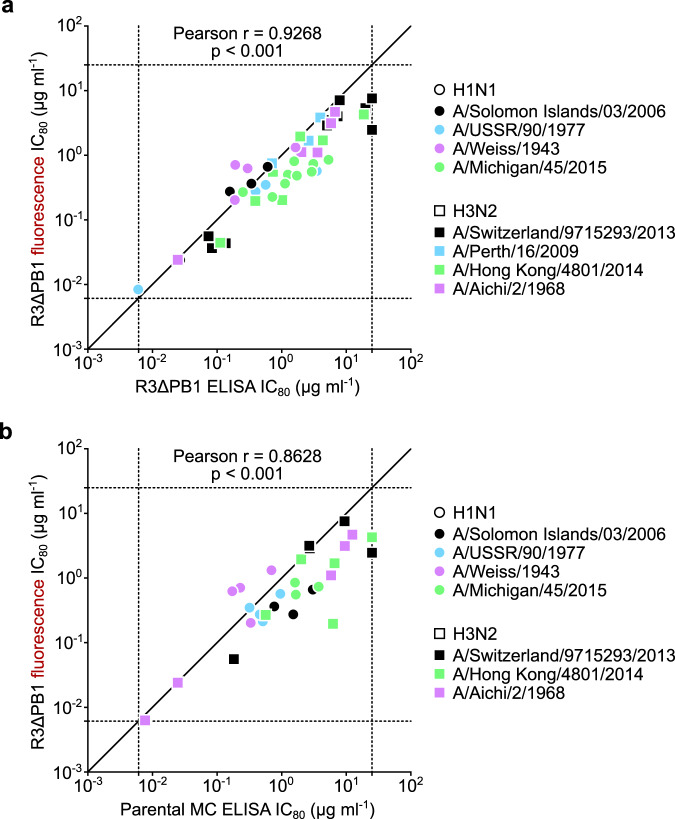
Assessment of R3∆PB1 virus for neutralization assay. **a** Correlation between fluorescence- and ELISA-based readouts. Each dot indicates the neutralization titers (IC_80_ µg ml^−1^) of a single monoclonal antibody against an R3∆PB1 influenza virus measured by fluorescence readout (*y*-axis) and ELISA with anti-NP antibody (*x*-axis). **b** Correlation between assays using R3∆PB1 and molecular clone (MC) viruses. Each symbol indicates the neutralizing activity (IC_80_ µg ml^−1^) of a single monoclonal antibody using R3∆PB1 (*y*-axis) and parental MC viruses (*x*-axis) expressing the same HA and NA. Colors indicate different viruses. Circles and squares represent H1N1 and H3N2 viruses, respectively. Dashed lines indicate upper and lower limits of detection. Pearson *r* correlation coefficient and two-tailed *p* values for each analysis are shown above each graph. Neutralization assays were performed at least twice and representative data are plotted.

We tested whether the R3∆PB1 viruses can be used in an influenza virus neutralization assay. To do so, neutralization assays were carried out utilizing both R3∆PB1 and parental viruses with matched HA and NA, where we compared the inhibitory concentration of a set of mAbs. To verify the reporter-based readout, the R3∆PB1 virus-infected cells were detected either by ELISA with anti-NP antibody or by fluorescence using an automated image-based plate reader at 24 h post infection. We measured neutralizing activity (80% inhibitory concentration, IC_80_) detected by both ELISA- and fluorescent reporter-based readouts for several mAbs against a total of 8 R3∆PB1 viruses (4 H1N1 and 4 H3N2 viruses) and found a strong positive correlation (Pearson *r* = 0.94, *p* < 0.001) between the two IC_80_ datasets, demonstrating that the fluorescent reporter-based readout yields comparable neutralization results to conventional ELISA-based detection method (Fig. 2a and Supplementary Fig. 3).

To assess the R3∆PB1 viruses with the MN assay format, we next determined neutralizing IC_80_ for 6 mAbs against matched pairs of R3∆PB1 and parental viruses (4 H1N1 and 3 H3N2 viruses). Cells infected with R3∆PB1 viruses were detected by fluorescence, while cells infected with parental viruses were detected by ELISA. The positive correlation (Pearson *r* = 0.87, *p* < 0.001) between neutralization IC_80_ of mAbs against R3∆PB1 and parental viruses shows that R3∆PB1 viruses retained the neutralization sensitivity of parental viruses with matched HA and NA (Fig. 2b). In conclusion, our results indicate that the neutralization assay using R3∆PB1 viruses with fluorescence-based readout can be used as high-throughput, safe, and reliable measurement of virus-neutralizing activity.

### Building a comprehensive panel of R3 viruses

We aimed to build a comprehensive panel of R3∆PB1 viruses spanning the entire antigenic evolution of human H1N1 and H3N2 subtype viruses. We selected representative influenza strains based on phylogenetic analysis of HA sequences deposited in public databases, literatures on genetic and antigenic evolution of human H1N1 and H3N2 influenza viruses, and vaccine strains utilized since 1930s^23,25–28^.

**Fig. 3:**
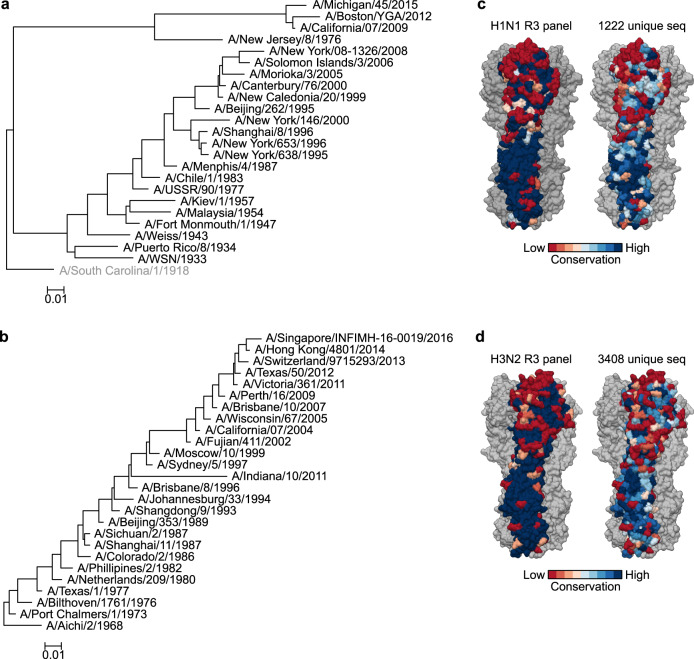
Phylogenetic and protein surface conservation analysis of HA of H1N1 and H3N2 viruses used in the study. **a**, **b** Phylogenetic tree of 24 H1 (**a**) and 26 H3 (**b**) HA sequences used in the R3 panel. The tree was generated by NJ method and rooted with A/South Carolina/1/1918 (**a**) (not used in the study) or with A/Aichi/2/1968 (**b**). Distance scale bar indicates 1% difference in nucleotide identity. **c**, **d** Protein surface conservation of H1 (**c**) and H3 (**d**) HA used in the R3 panel and a larger non-redundant representative dataset. Amino acid conservation at each residue was calculated and scored by using the Consurf server (https://consurf.tau.ac.il/) and rendered on the structure of A/California/07/2009 (PDB: 3LZG) (**c**) and A/Victoria/361/2011 (PDB:4WE8) (**d**). Conservation at each position was scored 1–9 (lowest to highest) and colored according to the conservation score on one protomer of HA trimer. Two other HA protomers are colored in gray.

H1N1 subtype virus was introduced into the human population in 1918 and circulated until it was replaced by H2N2 virus in 1957. H1N1 virus re-emerged in 1977 and circulated until 2009. During this period, H1N1 viruses evolved significantly through genetic drift into multiple clades with distinct genetic and antigenic properties^27,29–31^. To capture the antigenic variations of these viruses, we chose 7 matched HA and NA sequences from viruses circulating between 1933 and 1957 and 13 HA and NA sequences from viruses circulating between 1977 and 2009. The 2009 pandemic H1N1 virus acquired sustained human-to-human transmission and rapidly and completely replaced the pre-pandemic H1N1 virus. Since its emergence, the 2009 pandemic H1N1 virus has accumulated several amino acid substitutions linked to changes in antigenicity^32^. Therefore, we included 3 HA and NA sequences from viruses isolated between 2009 and 2015 and HA and NA sequences of swine-origin A/New Jersey/8/1976 (H1N1), which caused an isolated outbreak in 1976 in the United States. In summary, our H1N1 panel consists of 19 pre-pandemic strains, 3 pandemic strains, and 1 swine-origin H1N1 strain (Fig. 3a). Of note, our panel of H1N1 viruses includes genetically similar viruses to all World Health Organization (WHO)-recommended H1N1 vaccine strains between 1977 and 2015 (fludb.org/brc/vaccineRecommend.spg?decorator=influenza), antigenically representative viruses circulating in humans in the 1940s^31^, or representatives of the major HA and NA lineages of seasonal H1N1 viruses^30^.

H3N2 subtype virus has been circulating in humans since its emergence in 1968. Comprehensive genetic and antigenic analysis of human H3N2 viruses groups them into 14 distinct antigenic clusters^21,26,28,33^, and hence, we include several representatives from each antigenic cluster in our panel. Sporadic outbreaks with swine H3N2 variant (H3N2v) have also been described in humans^34^. Therefore, we included the swine-origin H3N2v strain, A/Indiana/10/2011. As a result, our H3N2 panel includes 25 human H3N2 strains and 1 H3N2v strain (Fig. 3b).

When we calculate the conservation of solvent-exposed surface among H1 and H3 HAs included in our panel, we notice that the head region of HA of both H1 and H3 is substantially more variable while the stem region is mostly conserved as expected (Fig. 3c, d). The degree of surface conservation reflects that of much larger datasets of H1 and H3 HAs (Fig. 3c, d), suggesting that the selected HA sequences in our R3 virus panel capture the HA diversity of human H1N1 and H3N2 viruses.

### Generation of R3∆HA viruses with highly pathogenic influenza virus sequences

Working with highly pathogenic influenza viruses (e.g., H5N1, H7N9, 1918 H1N1) or influenza lineages disappeared from human population (e.g., H2N2) requires high containment laboratories. Although R3∆PB1 viruses have limited capacity to replicate due to the requirement of PB1 complementation, they retain the ability to reassort the HA segment with wild-type influenza viruses. To prevent this event, we evaluated alternative approaches less susceptible to reassortment and generated reporter viruses expressing HA and NA of viruses with pandemic potential^19,35^.

First, we developed influenza viruses, unable to reassort HA segments, by making “rewired” R3∆PB1 viruses (R4∆PB1) as previously described^35^. For this purpose, two segments of influenza genome were altered: the PB1 segment was modified to encode HA, and the HA segment was modified to encode the tdKatushka2 reporter. Using these altered PB1 and HA segments and reverse genetics, we were able to rescue R4∆PB1 A/Switzerland/9715293/2013 (H3N2) virus in PB1-expressing cells (Supplementary Fig. 4a–c). By measuring neutralizing IC_80_ of 15 mAbs for both R3∆PB1 and R4∆PB1, we found a strong positive correlation (Pearson *r* = 0.94, *p* < 0.001) between IC_80_ neutralizing activity against the two viruses, demonstrating that these viruses have equivalent neutralization sensitivities (Supplementary Fig. 4d). Moreover, we found that the R4∆PB1 A/Switzerland/9715293/2013 (H3N2) virus did not reassort its HA segment with A/Solomon Islands/03/2006 (H1N1) when the two viruses were co-infected and propagated on PB1-expressing cells (Supplementary Fig. 4e). Although these results show that R4∆PB1 viruses can be safely rescued and used for high-throughput neutralization assay, further work is required to optimize the rescue and the propagation of R4∆PB1 to be a practical and deployable process for producing reporter viruses.

**Fig. 4:**
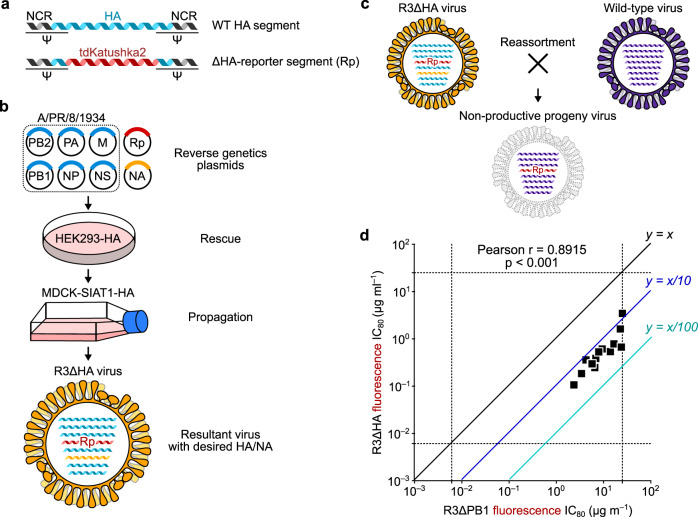
Development of R3∆HA influenza viruses. **a** Design of HA segment used to rescue R3∆HA virus. **b** R3∆HA virus requires cells expressing HA protein in trans for rescue and propagation. **c** Non-viable reassortment between R3∆HA and wild-type influenza viruses. Reassortant virus carrying the engineered HA segment encoding the reporter gene results in replication-deficient virus. Rp, tdKatushka2 reporter gene-encoded segment. **d** Neutralization sensitivity of R3∆HA viruses. Correlation between neutralization titers of 15 monoclonal antibodies against R3∆PB1 and R3∆HA of A/Switzerland/9715293/2013 H3N2 is shown. Each square indicates titers (IC_80_ µg ml^−1^) of a single monoclonal antibody against R3∆HA (*y*-axis) and matched R3∆PB1 viruses (*x*-axis). Dashed lines indicate upper and lower limits of detection. Neutralization assays were performed at least twice and representative data are plotted.

Next, we explored an alternative approach to rescue R3 viruses unable to reassort the HA segment by replacing the HA coding sequence with the reporter gene (R3∆HA). In this configuration, the virus lacks a functional HA segment and is unable to reassort the segment with other viruses (Fig. 4a–c), and it will be able to replicate only in HA-expressing cells^19^. To assess R3∆HA viruses for the reporter neutralization assay, we generated R3∆HA A/Switzerland/9715293/2013 (H3N2) virus to compare the neutralization IC_80_ of several mAbs against both R3∆PB1 and R3∆HA viruses. There was a positive correlation between neutralization IC_80_ determined with these viruses (Pearson *r* = 0.90, *p* < 0.001), although we noticed slight differences in sensitivity to neutralization when testing anti-stem antibodies against R3∆PB1 and R3∆HA viruses (Fig. 4d). Using this approach, we prepared 6 R3∆HA viruses (i.e., 2 H5N1 of clade 1, A/Vietnam/1203/2004, and clade 2.1.3, A/Indonesia/05/2005, 2 genetically and antigenically related H7N9, A/Anhui/01/2013 and A/Shanghai/02/2013, 1 H2N2, A/Singapore/1/1957, and 1 H10N8, A/Jiangxi-Donghu/346-2/2013) in addition to the 49 R3∆PB1 virus panel^36,37^.

### Characterizing influenza responses in serum samples from human vaccinees using R3 viruses

To assess neutralization activity in polyclonal sera using R3 viruses, we measured the neutralization titers in samples collected in the Phase I human clinical trials of experimental H5N1 and H7N9 vaccines conducted by the Vaccine Research Center in 2011 and 2015, respectively (NCT01086657 and NCT02206464, respectively). Briefly, healthy adult volunteers were immunized with DNA encoding H5 (A/Indonesia/05/2005) or H5N1 monovalent influenza vaccine (MIV), or DNA encoding H7 (A/Shanghai/02/2013) HA, H7N9 MIV, or combinations of H7 DNA and H7N9 MIV followed by a boost with H5N1 MIV or H7N9 MIV, respectively^38,39^. Neutralization titers were measured at the time of vaccination (week 0) and 2 weeks post-boost against vaccine-matched strain (i.e., H5N1 A/Indonesia/05/2005 or H7N9 A/Shanghai/02/2013 R3∆HA), two H1N1 R3∆PB1 (A/Michigan/45/2015 and A/New Caledonia/20/1999), and two H3N2 R3∆PB1 (A/Hong Kong/4801/2014 and A/Shangdong/9/1983) viruses. For individuals vaccinated with the H5 prime-boost regimen^38^, we found that neutralization titers against the vaccine strain H5N1 A/Indonesia/05/2005 increased at 2 weeks post-boost from a baseline reciprocal IC_80_ GMT of 727.8 [range 206.6–1603.5] to a final reciprocal IC_80_ GMT of 33,014.2 [range 3235.7–223,905.4] (*p* < 0.0001). We also observed a significant increase (*p* = 0.0003) in the reciprocal IC_80_ titers against H1N1 A/Michigan/45/2015 virus (Supplementary Fig. 5a). Similarly, for individuals vaccinated with the H7 prime-boost regimen^39^, we found that titers against H7N9 A/Shanghai/02/2013 virus had increased significantly upon vaccination (from a baseline reciprocal IC_80_ GMT of 246.9 [range <40–1192.1] to 3134 [range 765.2–31,285.8] (*p* = 0.0013). Of note, the H7 prime-boost regimen did not increase significantly neutralization titers against two H1N1 nor two H3N2 viruses tested in any of these individuals (Supplementary Fig. 5b). Together, these results establish that the neutralization assay using R3 viruses can be used to measure the influenza virus-neutralizing activities in human polyclonal sera.

### Measurement of the viral inhibitory activity of anti-NA antibodies using R3 influenza virus

To assess the viral inhibitory activity of anti-NA antibodies, we adapted our assay using the R3 viruses by adding a thixotropic overlay and quantifying the virus-infected area (i.e., fluorescent plaques). Broadly cross-protective anti-NA mAb 1G01^40^ and murine anti-NA mAb CD6^41^ inhibited virus propagation in a concentration-dependent manner while non-targeting mAb D25 (anti-respiratory syncytial virus)^42^ had no effect on propagation of R3∆PB1 A/California/07/2009 H1N1 viruses (Supplementary Fig. 6). These results suggest that our fluorescent plaque-reduction assay with influenza R3 viruses allows live, high-throughput measurement of viral inhibitory activities targeting NA and will facilitate the characterization of novel vaccine candidates and mAbs with anti-NA responses.

### Profiling of influenza neutralizing mAbs with a 55-virus panel

**Fig. 5:**
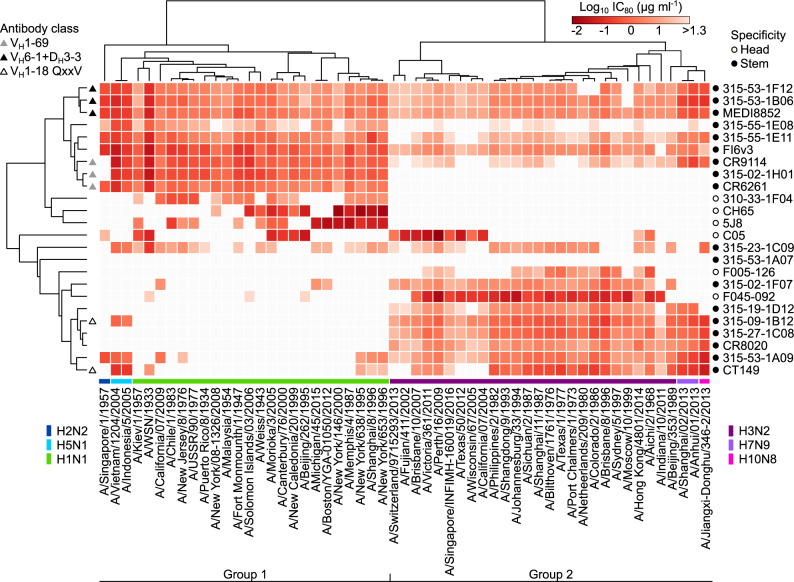
In-depth neutralization profiling of influenza monoclonal antibodies. Heatmap of neutralization titers (IC_80_ μg ml^−1^) of 24 monoclonal antibodies against a panel of 55 R3 influenza A viruses. Virus subtypes are color-coded and indicated at the bottom of the heatmap. Open and closed circles indicate specificity of monoclonal antibodies and are shown on the right of the heatmap (open, HA head-; closed, HA stem-directed). Convergent classes of bnAbs with specific gene signatures are indicated on the left of the heatmap as triangles (gray, V_H_1-69; black, V_H_6-1 + D_H_3-3; open, V_H_1-18 QxxV). Heatmap and unsupervised clustering was made with the ClustVis server (https://biit.cs.ut.ee/clustvis/). Note: H1N1 and H3N2 viruses are R3∆PB1 and all other viral subtypes are R3∆HA viruses. Neutralization assays were performed at least twice and representative data are plotted.

Using our comprehensive panel of R3 influenza viruses spanning 6 different influenza A subtypes, we profiled the neutralization breadth and potency of a total of 24 human mAbs. Thirteen of them were isolated at the Vaccine Research Center from peripheral blood mononuclear cells collected as part of the H5N1 or H7N9 vaccine clinical trials^39,43–45^, while 11 other antibodies were previously described elsewhere^46–55^. Among 24 antibodies, 18 antibodies recognize epitopes on the conserved HA stem region and 6 antibodies (i.e., CH65, 5J8, C05, F045-092, F005-126, and 310-33-1F04) bind within the HA head region. HA stem-binding 315-53-1A07 which did not neutralize any of the viruses was included in our panel. Of note, neutralization IC_80_ values of the mAbs did not change drastically between 18 and 24 h after infection while the virus-infected cells dramatically increased during this time period (Supplementary Fig. 7), providing a reasonable flexibility for assay operation and fluorescent readout. We generated a matrix of neutralizing profiles for 24 antibodies against 55 R3 viruses consisting of 1320 data points and analyzed the matrix by hierarchical clustering (Fig. 5). Despite the fact that there is no associated information about viruses in the input dataset, the profile matrix segregates not only groups of viruses but also virus subtypes into defined clusters (Fig. 5). Interestingly, the hierarchical clustering groups several antibodies according to their immunogenetic composition or convergent antibody class^44,56,57^. For example, MEDI8852 is clustered with 2 other antibodies (315-53-1F12 and 315-53-1B06) and all the three antibodies belong to the V_H_6-1 + D_H_3-3 convergent multi-donor class^57^, while CR9114 is clustered with CR6261 and 315-02-1H01, which all shares V_H_1-69. The latter case is particularly noteworthy as CR9114 has much broader neutralizing capacity than the other two, yet clustered with the stereotypical group 1-specific V_H_1-69 antibodies CR6261 and 315-02-1H01 (Fig. 5).

**Fig. 6:**
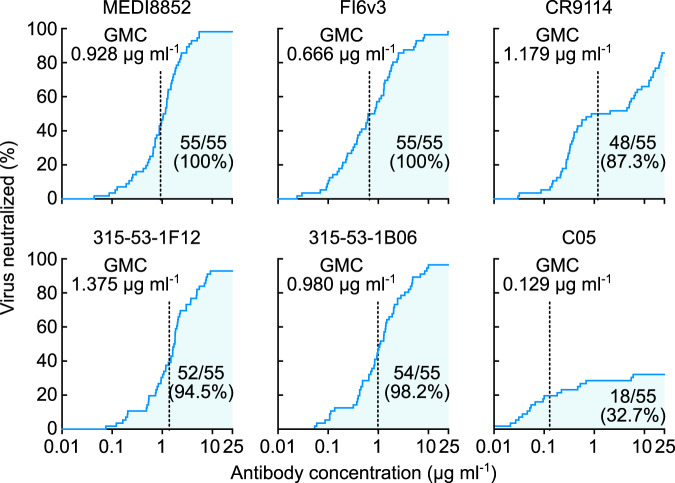
Neutralization breadth-potency analysis of broadly cross-reactive stem-directed antibodies and highly potent receptor-binding site antibody. Neutralization titers (IC_80_ µg ml^−1^) against a panel of 55 R3 viruses were used to generate the breadth-potency plot for each antibody. Geometric mean IC_80_ concentration (GMC) of each antibody was calculated only for the viruses neutralized by given antibody and indicated as a vertical dashed line on each graph. Shaded areas correspond to the number of viruses neutralized by given antibody. Both the number of viruses neutralized out of 55 R3 viruses and neutralization coverage (%) are indicated on each graph.

Deep characterization of neutralizing profiles also predicts the developmental pathway for each bnAb. The convergent multi-donor bnAbs in V_H_1-18 QXXV class (i.e., 315-09-1B12 and CT149) neutralize many group 2 viruses while having very limited breadth against group 1 viruses (Fig. 5). This confirms previous findings in which the unmutated common ancestor (UCA) of this class of bnAbs engages only group 2 HAs and acquires group 1 reactivity through somatic hypermutation^57^. Conversely, the V_H_6-1 + D_H_3-3 class bnAbs (i.e., MEDI8852, 315-53-1F12, and 315-53-1B06) possess higher neutralization potency against group 1 viruses than group 2 viruses (Fig. 5), and this is consistent with the preferential engagement of group 1 HAs to the UCAs of this class^57^. This deep neutralization profiling dataset also allows us to generate neutralization breadth–potency curves at relatively high resolution (Fig. 6). Previous studies used a dataset generated by pseudotyped lentiviral neutralization assays with 15–17 selected HA–NA sequences^45,57^ and provided a limited understanding of neutralization breadth both because of the relatively small number of strains and the hypersensitivity of the pseudotyped lentivirus assay format. By determining the neutralization profiles using our comprehensive R3 virus panel, we found two antibodies (MEDI8852 and FI6v3) that were capable of neutralizing all 55 viruses (Fig. 6). The other two V_H_6-1 + D_H_3-3 class mAbs, 315-53-1F12 and 315-53-1B06, neutralized 52 (94.5%) and 54 (98.2%) of 55 viruses, respectively. Although CR9114 neutralized 48 out of 55 viruses (87.3%) its breadth–potency plot demonstrated a biphasic curve, highlighting the preferential neutralization of group 1 viruses (the first phase) and lower potency against group 2 viruses (the second phase) by this antibody (Fig. 6). The RBS-directed antibody C05 showed limited neutralization breadth (32.7%) yet was extremely potent (Fig. 6).

Overall, the neutralization profiling performed with the reporter influenza virus assay provides less biased breadth and potency information than the pseudotyped lentivirus assay or highly variable traditional MN assay. High-resolution information may also help predict the class and the origin of antibodies with particular neutralization signatures.

## Discussion

Current influenza vaccines are imperfect and there is a large room to improve their efficacy, consistency, and breadth. Historically, efficacy has been associated with serum HAI activity^58,59^ and the HAI assay was developed as a surrogate for virus neutralization^60,61^. To improve current vaccines and to develop new vaccine concepts that will have broader and more durable efficacy, accurate measurement of neutralizing activity across a broad range of influenza viruses is critical. The HAI assay is a surrogate only for neutralization directed at the RBS on HA, therefore neutralization targeting the HA stem or other relatively conserved sites on HA and NA will not correlate with HAI. In addition, some new drifted seasonal strains of influenza and subtypes with pandemic potential do not always have predictable hemagglutination properties. Therefore, to encompass the variety of HA and NA strains and the antibodies induced by diverse vaccine approaches, emphasis on a reproducible and authentic measurement of virus-neutralizing activity is needed.

Unlike the HAI assay that measures the antibody’s ability to block receptor binding of viral HA, plaque reduction^62^ and MN^63^ assays can capture neutralizing antibodies that block any virus replication step, including attachment, internalization, pH-induced conformational change of HA, membrane fusion, and virus egress. Despite efforts initiated by WHO, execution of influenza neutralization assays is still not standardized, leading to significant variability among results reported by different laboratories^9,63^. Development of a large panel of representative influenza viruses and significant improvement of assay throughput and safety are highly anticipated in the field and will enable deep characterization and systematic comparisons of influenza virus-neutralizing antibody responses. The virus panel combined with serum and mAb standards will allow normalization across assay platforms and facilitate the evaluation of universal influenza vaccine candidates. In the present study, we address these outstanding needs by building an engineered influenza reporter virus-based neutralization assay coupled with high-throughput image-based readout in a biosafety level 2 setting. Our panel consists of 55 reporter viruses capturing almost the entire antigenic evolution of human H1N1 and H3N2 subtypes as well as historical human H2N2 and three other subtypes circulating at the human–animal interface. Moreover, we were able to rescue and propagate R3 chimeric influenza viruses encoding the HA and NA of influenza B using the internal genes of A/WSN/1933 influenza A virus. R3∆PB1 chimeric influenza A/B viruses can replicate only in cells expressing influenza A PB1 in trans. This approach to rescue viruses expressing HA and NA of influenza B viruses was described in several previous reports^64–66^, and it was shown that chimeric influenza A/B viruses have similar growth profiles as wild-type influenza B viruses^66^. Although none of the 24 antibodies included in our study neutralized these R3∆PB1 chimeric influenza A/B viruses, we have successfully utilized these viruses in neutralization assays to assess anti-influenza B virus-neutralizing antibody responses elicited by experimental vaccines^67^.

Direct detection of fluorescent reporter allowed us to measure virus replication in live cells without additional signal amplifications unlike ELISA- or hemagglutination-based readout. Image-based readout was chosen to further facilitate fast and precise data acquisition and analysis. In contrast to ELISA-based assays, in which the signal is measured for the entire well after a series of signal amplification steps, image-based detection provides higher signal-to-noise ratio, dynamic range, and precision at single-cell resolution. The R3 virus neutralization assays can be readily automated to minimize hands-on time and human errors. It is also worth noting that the fluorescent image-based readout makes the assay more cost-effective compared with other assays such as ELISA- and luciferase-based assays. By using R3 viruses, we can detect virus-inhibitory activity of both anti-HA and anti-NA antibodies by counting number of fluorescent cells or measuring size of fluorescent plaques in a safe and high-throughput manner.

Recent attempted improvements of the WHO-recommended ELISA-based MN assay protocol^68–70^ underscore the need for the development of more reliable assays. As noted, previous reports have described the potential utility of R3∆PB1^18^ and R3∆HA^19,20^ influenza viruses in neutralization assays. However, these efforts did not lead to widespread adoption and the development of standardized influenza neutralization assays due to the small number of available reporter viruses, impediments to access essential reagents (e.g., influenza reverse genetics plasmids and stable cell lines), and lack of comprehensive validation. We found that the antigenicity and neutralization sensitivity of R3∆PB1 is similar to that of authentic influenza viruses, while R3∆HA viruses appeared to be slightly more sensitive to neutralization than R3∆PB1. This is likely due to less efficient HA incorporation into R3∆HA viruses^19,20^ and represents a common feature of R3∆HA viruses. Although we noticed that the neutralization sensitivity of R4∆PB1 closely matched that of R3∆PB1 implying their potential superiority for building viruses that are unlikely to reassort, additional optimization of R4∆PB1 virus is necessary to become a practical alternative due to its poor rescue efficiency. Although the use of R3 (and R4) viruses offers further advancements in the assay throughput, safety, and precision, this approach has its own limitations. Since the reporter segment is not required for viral replication, it is inevitable that after extensive virus passage, the reporter segment may accumulate mutations made by the error-prone viral RNA polymerase, which in turn, will diminish reporter gene activity and/or expression. However, it is possible to prepare virus stocks relatively quickly with five or less passages, and we confirm that these stocks retain an active reporter gene expression. Additionally, genomic stability of R3 influenza viruses can be improved by utilizing the variant PB1 polymerase with lower error rates as reported recently^71^ and designing R3 viruses capable of replicating only when the engineered genomic segment is functional, such as inducible gene-expression systems.

As we continue to expand our collection of viruses and neutralizing antibodies, we will be able to perform neutralization fingerprint analysis^72^ to provide a better understanding of the relationships between fine epitope specificity and neutralization breadth and potency and allow computational predictions of the epitope-specific contributions of polyclonal serum antibodies to overall neutralizing activity. This type of analysis will foster the immune monitoring of antibody responses elicited by universal influenza vaccine candidates, particularly those targeting the conserved HA stem supersite^73–75^. By describing our methods and depositing sequences required to generate R3 viruses, we provide the basis for implementing this technology for the strains reported here as well as for future emerging viruses. We anticipate that our assay will foster rapid discovery and characterization of influenza bnAbs as well as virus-inhibitory antibodies and facilitate efficient evaluation of vaccine-elicited antibody responses for accelerating the efforts to develop universal influenza vaccines.

## Methods

### Plasmids

To prepare influenza reverse genetics plasmids for rescue of influenza A H1N1 or H3N2 viruses described in this study, HA and NA coding sequences were retrieved from Genbank; NCRs of A/WSN/1933 for H1N1 or A/Netherlands/009/2010 for H3N2 viruses were added at both ends. Full-length HA and NA sequences were cloned into a dual promoter influenza reverse genetics plasmid previously described^76^. To rescue influenza viruses, dual promoter plasmids encoding internal genes of A/WSN/1933 (pHW181-PB2, pHW182-PB1, pHW183-PA, pHW185-NP, pHW187-M, pHW188-NS) were used^76^. To prepare PB1 reporter segment used to rescue R3 viruses, the sequence containing the PB1 genome packaging signals of A/WSN/1933^22^ and mKate2 or tdKatushka2 reporter coding region^23^ (Addgene Cat No. 56049) was synthesized and cloned using BsmBI (New England Biolabs) restriction sites into the dual promoter influenza reverse genetics plasmid. Similarly, HA reporter segment was synthesized with HA genome packaging signals of A/Puerto Rico/1934^19^ flanking the tdKatushka2 reporter sequence and cloned into the dual promoter influenza reverse genetics plasmid using BsmBI restriction sites. To prepare the influenza reverse genetics plasmid using chicken beta-actin CAG pol-II promoter (Addgene Cat No. 41583), an insert comprising human pol-I promoter and mouse pol-I terminator sequences in negative orientation flanking two BsmBI restriction sites was cloned using KpnI and XhoI restriction sites. Then full-length of influenza genes of high-yield A/Puerto Rico/8/1934^77^ were cloned into BsmBI restriction sites. To prepare plasmids for stable cell line development, sequences of Streptomyces puromycin *N*-acetyl-transferase (PAC), which confers resistance to puromycin, followed by self-cleaving peptide of Thosea asigna virus 2A (T2A) and coding region of influenza A PB1, which has PB1-F2 transcript unmodified, or HA genes were synthesized and cloned into pCAGGS plasmid (Addgene Cat No. 41583) using *Kpn*I and *Xho*I restriction sites. All plasmids were confirmed by Sanger sequencing.

### Cells

To propagate influenza viruses, MDCK-SIAT1 cells (Millipore Sigma) were used. Cells were maintained with complete media comprising Dulbecco’s modified Eagle’s medium high glucose (DMEM; ThermoFisher) supplemented with 10% (v/v) heat-inactivated fetal bovine serum (Gemini Bio-Products), 100 units ml^−1^ penicillin (ThermoFisher), 100 μg ml^−1^ streptomycin (ThermoFisher), and geneticin (1 mg ml^−1^) (ThermoFisher). To develop constitutively PB1- or HA-expressing MDCK-SIAT1 cells, one plasmid encoding both puromycin resistance and influenza genes was transfected into MDCK-SIAT1 cells using Lipofectamine 2000 (ThermoFisher). Two days post transfection, cells were transferred from 6-well plates into 10-cm dishes containing DMEM media with 10% bovine serum (Gemini Bio-Products), penicillin, streptomycin, geneticin, and puromycin (0.25 μg ml^−1^; ThermoFisher) for selection. Medium was changed every 48 h. Clonal selection was performed using 8 or 10 mm cloning cylinders (Fisher Scientific) about 2 weeks after the transfection. Clonal cell lines were screened using a reporter virus prepared on a polyclonal cell line. To rescue influenza viruses, Flp-In 293 cells (ThermoFisher) were transfected as described below.

### Reverse genetics of influenza viruses

For molecular clone influenza viruses, an eight-plasmid approach in which each influenza segment is inserted between pol-II (positive orientation) and pol-I (negative orientation) promoters was used to rescue parental molecular clone viruses. Briefly, Flp-In 293 cells were transfected with dual promoter plasmids encoding each influenza segment of A/WSN/1933 (obtained from St. Jude Children’s Research Hospital) and a CMV-driven plasmid expressing human transmembrane serine protease 2 (hTMPRSS2). Transfection was performed using Lipofectamine 3000 in 6-well plates coated with D-lysine. Three days post infection, TPCK-treated trypsin (0.5 μg ml^−1^, Sigma) was added to the transfected cells for 2–4 h. Supernatant was harvested, cleared by centrifugation (200 × *g*, 10 min), and used for propagation in MDCK-SIAT1 cells by limiting dilution. For virus propagation, the inoculum was prepared in virus growth medium comprising of OptiMEM (ThermoFisher) supplemented with TPCK-treated trypsin at 1 μg ml^−1^. MDCK-SIAT1 flasks containing cells at ~80% confluence were washed twice with phosphate-buffered saline (PBS) and incubated with the inoculum at 37 °C for virus adsorption. After 1 h, the inoculum was removed, cells were washed with PBS, and virus growth media was added to the cells. The infected cells were incubated at 37 °C in a humidified 5% CO_2_ atmosphere. After 2 days, supernatant was harvested when cytopathic effect reached 40–60%, cleared by centrifugation (200 × *g*, 10 min), aliquoted, and stored at −80 °C. For influenza HA and NA sequencing, viral RNA was extracted with the RNeasy Extraction Kit (Qiagen). Influenza viral RNA was amplified using the Qiagen One-Step RT-PCR Kit (Qiagen) and HA- and NA-specific primers tagged with M13 sequences. Each amplicon was sequenced with M13 primers in both directions. Primer sequences are available upon request. Sequences were analyzed using Sequencer 5.4 (Gene Codes).

For R3∆PB1 influenza viruses, Flp-In 293 cells were transfected with eight dual promoter influenza reverse genetics plasmids (the PB1 reporter segment plasmid replaced the plasmid encoding PB1 of A/WSN/1933) together with pol-II-driven expression plasmid encoding PB1 of A/WSN/1933 and hTMPRSS2. Rescued viruses were propagated in MDCK-SIAT1 cells constitutively expressing PB1 of A/WSN/1933 in the presence of TPCK-treated trypsin (1 μg ml^−1^). Virus stocks were stored at −80 °C.

For replication-restricted rewired ∆PB1 influenza viruses, Flp-In 293 (ThermoFisher) cells were transfected with eight dual promoter influenza reverse genetics plasmids (PB1 segment comprises PB1 genome packaging signals flanking the coding region of an HA segment in which the HA genome packaging signals have been destroyed by introducing silent mutations, while the HA segment of R4∆PB1 contains the reporter gene flanked by intact HA genomic packaging signals) together with pol-II-driven expression plasmid encoding PB1 of A/WSN/1933 and hTMPRSS2. Rescued viruses were propagated as described above.

For R3∆HA influenza genes, Flp-In 293 cells were transfected with eight influenza reverse genetics plasmids encoding sequences of high-yield A/Puerto Rico/8/1936 (HA segment was replaced with HA reporter segment described above), pol-II-driven HA and hTMPRSS2 expressing plasmids. Viruses were propagated in MDCK-SIAT1 cells constitutively expressing influenza HA gene in the presence of TPCK-treated trypsin (1 μg ml^−1^). Virus stocks were stored at –80 °C

### Phylogenetic and evolution-based conservation analyses of influenza HA

Nucleotide sequences of mature H1 HA (*N* = 25) and H3 HA (*N* = 26) proteins were aligned using Muscle algorithm found in Bioedit v7.2.5. Phylogenetic trees were generated using neighbor-joined methods and Kimura 2-parameter substitution model as implemented in MEGA v10. Evolution-based conservation analyses of amino acid residues in the extracellular region of H1 and H3 HA proteins was done using Consurf (http://consurf.tau.ac.il) and visualized on the atomic structures of HA proteins of A/California/07/2009 (PDB ID: 3LZG) and A/Victoria/361/2011 (PDB ID: 4WE8). To evaluate the conservation of each amino acid of H3 HA proteins isolated from influenza viruses circulating in humans, full-length H3 HA sequences of human H3N2 viruses were obtained from the GISAID database (http://platform.gisaid.org). Alignment of nucleotide sequences was performed using MAFFT v7 server-based algorithm using default settings (https://mafft.cbrc.jp/alignment/server/large.html). After removal of redundant sequences and sequences with gaps or degenerate nucleotide bases, we obtained a dataset of 16,893 unique sequences. Due to the large size of this sequence dataset, we clustered sequences (CD-HIT EST at http://weizhong-lab.ucsd.edu/cdhit-web-server/cgi-bin/index.cgi?cmd=cd-hit-est) with identity >99.6% and choose a representative sequence from each cluster. The final dataset used to estimate evolution-based conservation of amino acid residues in human H3 HA proteins had 3408 sequences. Similarly, an alignment of 1222 H1 HA of H1N1 viruses circulating in humans between 1918 and 2019 sequences was used to evaluate the conservation of each amino acid of this protein.

### Virus titration and neutralization

For molecular clones, ELISA-based influenza MN assay was performed following WHO-recommended protocol. Briefly, influenza viruses were titrated in MDCK-SIAT1 cells plated in 96-well plates at 50,000 cells ml^−1^ 24 h before infection. Virus dilutions were prepared using OptiMEM supplemented with TPCK-treated trypsin (1 µg ml^−1^). Infected cells were incubated at 37 °C in a humidified 5% CO_2_ atmosphere. After 18 h, cells were fixed with 80% cold acetone and air dried. Virus replication was detected by biotin-conjugated antibodies to influenza virus nucleoprotein (MAB8257B and MAB8258B, Millipore Sigma) and was visualized with horseradish peroxidase-conjugated streptavidin and SureBlue TMB Microwell Peroxidase Substrate (KPL). Absorbance was read at 450 nm (A450) and 650 nm (A650) with the SpectraMax Paradigm microplate reader (Molecular Devices). The A650 was used to subtract plate background. Half-maximal tissue culture infectious dose (TCID_50_) titer was calculated using Reed–Muench method. Neutralization assays were performed using 100–200 TCID_50_ units of virus and 4-fold antibody dilutions made in OptiMEM supplemented with TPCK-treated trypsin. Virus and antibody were mixed in equal volumes and incubated 1 h at 37 °C prior to adding to substrate MDCK-SIAT1 cells. Control wells of virus alone (VC) and diluent alone (CC) were included on each plate. Fifty microliters of antibody–virus mixture were then added to wells of pre-washed cells in duplicate and the plates were incubated for 18 h at 37 °C in a humidified 5% CO_2_ atmosphere. Infected cells were detected as described above. The percent neutralization was calculated by constraining the VC control as 0% and the CC control as 100% and plotted against antibody concentration. A curve fit was generated by a four-parameter nonlinear fit model in Prism (GraphPad). The 80% (IC_80_) inhibitory concentrations were obtained from the curve fit for each antibody.

Titer of R3∆PB1 or R3∆HA viruses was measured in PB1-expressing MDCK-SIAT1 cells plated in 96-well black plates with transparent bottom (Greiner) at 18 h post infection and counting fluorescent foci using Celigo Image Cytometer (Nexcelom) with customized red channel to enhance detection of mKate2/tdKatushka2 reporter (EX 540/80 nm, DIC 593 nm and EM 593/LP nm). In Celigo operation and analysis software v4.1, Target 1 protocol was used to detect and count fluorescent foci. Titer was expressed as fluorescent foci per ml. For each neutralization reaction, virus dilution that resulted in cca. 1000 (500–4000) fluorescent foci per well at 18 h post infection was used. Neutralization assays using R3 viruses performed to compare the fluorescence- and ELISA-based assays were done in 96-well black transparent bottom plates. Cells were plated 24 h before the experiment. Neutralization reaction was done as described above. R3 influenza neutralization assay was optimized to be performed in 384-well plate format. PB1-expressing MDCK-SIAT1 cells were washed twice with PBS, re-suspended in OptiMEM, and plated 2 h before the assay in 384-well plates at 150,000 cells ml^−1^ (20 µl per well). Twenty-five microliters of each neutralization mixture consisted of 2 μg ml^−1^ TPCK-treated trypsin and equal parts of virus and 4-fold serial dilutions of antibodies were transferred to wells in quadruplicate. Control wells of virus alone (VC) and diluent alone (CC) were included on each plate. Fluorescent foci were counted at 18–24 h post infection using a Celigo instrument. Neutralization titers were calculated using Prism as described above.

Detailed protocols are provided as Supplementary Notes 1 and 2.

### Antibody preparation

Sequences of immunoglobulin heavy and light chains were synthesized and cloned into human IgG1 as previously described^49^. The expression vectors were transiently transfected into Expi293F (ThermoFisher) using ExpiFectamine 293 transfection reagents (ThermoFisher). mAbs were purified using sepharose Protein-A (GE Healthcare) following the manufacturer’s instructions.

### Negative stain EM

Virus preparations were mixed at a 1:1 ratio with fixative containing 4% glutaraldehyde and 0.2 M cacodylate buffer, pH 7. A drop of the fixed sample was placed on a carbon-coated, glow-discharged copper grid for about 30 s. The drop was then removed using filter paper, and the grid was washed with three drops of buffer containing 10 mM HEPES, pH 7, and 150 mM NaCl, followed by negative staining with 0.75% uranyl formate. Imaging was performed using a ThermoFisher Talos F200C electron microscope operated at 200 kV and equipped with a Ceta camera.

### Statistics and reproducibility

All statistical analysis was performed using the GraphPad Prism software. Specific tests used to determine statistical significance are indicated in the “Methods” section and corresponding figure legends. Probability values <0.05 were considered statistically significant. All the figures are compiled using Inkscape (v1.0.0).

### Reporting summary

Further information on research design is available in the Nature Research Reporting Summary linked to this article.

## Supplementary information

Supplementary Information

Reporting Summary

## Data Availability

All images and data were generated and analyzed by the authors and will be made available by the corresponding authors (B.S.G. and M.K.) upon reasonable request. Influenza reverse genetics plasmids were obtained from St. Jude Research Hospital thorough an MTA. All sequences corresponding to the influenza HA, NA, and reporter segments used in the present study have been deposited to NCBI Genbank under accession numbers MW298159–MW298274. A reporting summary for this article is available as a Supplementary Information file. Source data are provided with this paper.

## Source data

Source Data
