# Supplementary material for: A comprehensive influenza reporter virus panel for high-throughput deep profiling of neutralizing antibodies
Source: Nat Commun. 2021 Mar 19;12:1722. doi: 10.1038/s41467-021-21954-2 (PMC7979723; doi:10.1038/s41467-021-21954-2)
Supplement: Supplementary file 1 — Supplementary Information [file 41467_2021_21954_MOESM1_ESM.pdf]

Supplementary Information for

A comprehensive influenza reporter virus panel for high-throughput deep profiling of neutralizing antibodies

Adrian Creanga[1], Rebecca A. Gillespie[1], Brian E. Fisher[1], Sarah F. Andrews[1], Julia Lederhofer[1], Christina Yap[1], Liam Hatch[1], Tyler Stephens[2], Yaroslav Tsybovsky[2], Michelle C. Crank[1], Julie E. Ledgerwood[1], Adrian B. McDermott[1], John R. Mascola[1], Barney S. Graham[1]*, Masaru Kanekiyo[1]*

[1]Vaccine Research Center, National Institute of Allergy and Infectious Diseases, National Institutes of Health, Bethesda, MD 20892, United States

[2]Electron Microscopy Laboratory, Cancer Research Technology Program, Frederick National Laboratory for Cancer Research sponsored by the National Cancer Institute, Frederick, MD 21702, United States

*Correspondence to: bgraham@nih.gov (B.S.G.) or kanekiyom@nih.gov (M.K.)

Table of contents:

Supplementary Fig. 1 2
Supplementary Fig. 2 3
Supplementary Fig. 3 4
Supplementary Fig. 4 5
Supplementary Fig. 5 7
Supplementary Fig. 6 8
Supplementary Fig. 7 10
Supplementary Table 1 12
Supplementary Note 1 13
Supplementary Note 2 30
Supplementary Methods 37
Supplementary References 39

5'-
*agcgaaagcaggcaaaccatttga*TtggTtgtcaatccgactttacttttcttaaaagtgccagcacaaaTtgctataagcacaactttcccttatactggagaccctccttacagccTtgggacaggaacaggatac
accTtgggtaccATGGTGGGTGAGGATAGCGTGCTGATCACCGAGAACATGCACATGAAACTGTACATGGAGGGCACCGTGAACGACCACCACTTCAA
GTGCACATCCGAGGGCGAAGGCAAGCCCTACGAGGGCACCCAGACCATGAAGATCAAGGTGGTCGAGGGCGGCCCTCTCCCCTTCGCCTTCGA
CATCCTGGCTACCAGCTTCATGTACGGCAGCAAAACCTTTATCAACCACACCCAGGGCATCCCCGACTTCTTTAAGCAGTCCTTCCCTGAGGGCTT
CACATGGGAGAGGATCACCACATACGAAGACGGGGGCGTGCTGACCGCTACCCAGGACACCAGCCTCCAGAACGGCTGCCTCATCTACAACGTC
AAGATCAACGGGGTGAACTTCCCATCCAACGGCCCTGTGATGCAGAAGAAAACACTCGGCTGGGAGGCCAGCACCGAGATGCTGTACCCCGCTG
ACAGCGGCCTGAGAGGCCATGCCCAGATGGCCCTGAAGCTCGTGGGCGGGGGCTACCTGCACTGCTCCCTCAAGACCACATACAGATCCAAGAA
ACCCGCTAAGAACCTCAAGATGCCCGGCTTCTACTTCGTGGACAGGAGACTGGAAAGAATCAAGGAGGCCGACAAAGAGACCTACGTCGAGCAG
CACGAGATGGCTGTGGCCAGGTACTGCGACCTGCCTAGCAAACTGGGGCACAGCGGGCATGGCACCGGCAGCACCGGCAGCGGCAGCTCCGG
TACtGCCTCCTCCGAGGACAACAACATGGCCATGGTGGGTGAGGATAGCGTGCTGATCACCGAGAACATGCACATGAAACTGTACATGGAGGGCAC
CGTGAACGACCACCACTTCAAGTGCACATCCGAGGGCGAAGGCAAGCCCTACGAGGGCACCCAGACCATGAAGATCAAGGTGGTCGAGGGCGG
CCCTCTCCCCTTCGCCTTCGACATCCTGGCTACCAGCTTCATGTACGGCAGCAAAACCTTTATCAACCACACCCAGGGCATCCCCGACTTCTTTAA
GCAGTCCTTCCCTGAGGGCTTCACATGGGAGAGGATCACCACATACGAAGACGGGGGCGTGCTGACCGCTACCCAGGACACCAGCCTCCAGAAC
GGCTGCCTCATCTACAACGTCAAGATCAACGGGGTGAACTTCCCATCCAACGGCCCTGTGATGCAGAAGAAAACACTCGGCTGGGAGGCCAGCA
CCGAGATGCTGTACCCCGCTGACAGCGGCCTGAGAGGCCATGCCCAGATGGCCCTGAAGCTCGTGGGCGGGGGCTACCTGCACTGCTCCCTCA
AGACCACATACAGATCCAAGAAACCCGCTAAGAACCTCAAGATGCCCGGCTTCTACTTCGTGGACAGGAGACTGGAAAGAATCAAGGAGGCCGAC
AAAGAGACCTACGTCGAGCAGCACGAGATGGCTGTGGCCAGGTACTGCGACCTGCCTAGCAAACTGGGGCACAGCTCCGGACTCAGATCTTGAct
cgaggtttccagagcccgaattgatgcacgaattgatttcgaatctggaaggataaagaaagaggagttcactgagatcatgaagatctgttccaccattgaagagctcagacggcaaaaatag*tgaatttagcttgtc*
cttcatgaaaaaatgccttgtttctact-3'

Italics: NCR
Red: TdKatushka2 ORF
Underlined: restriction sites (KpnI/XhoI)
Capital letters (black): mutated ATG in the coding packaging region

Supplementary Fig.1. Sequence of PB1 segment of R3ΔPB1 influenza viruses. Sequences corresponding to tdKatushka2 reporter is in red, non-coding regions are italicized, coding region of the packaging signals are in black, mutated ATG start codons in the 5' PB1 packaging signal are indicated with capital letters, restriction sites are underlined.

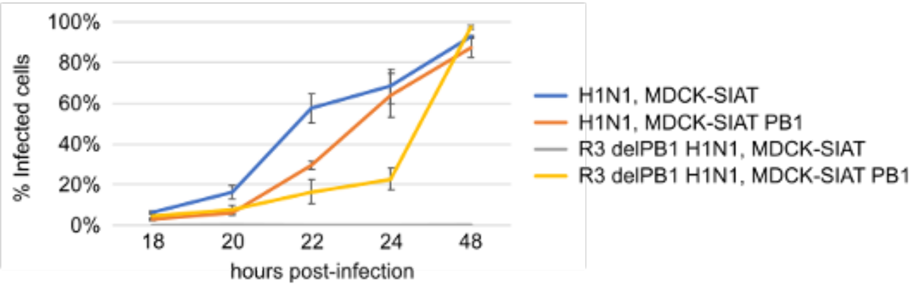

Supplementary Fig. 2. Growth kinetics of R3ΔPB1 virus in cells with or without PB1 expression. MDCK-SIAT1 and PB1-expressing MDCK-SIAT1 were infected with R3ΔPB1 or the parental A/Michigan/45/2015 (H1N1) viruses. Infected cells fixed with 4% paraformaldehyde and permeabilized with 0.01% Triton-X were detected by ELISA with biotin-conjugated antibodies to influenza virus nucleoprotein and imaged with streptavidin coupled with Alexa488. DAPI (300 nM) was used to label nuclei of all cells. Stained cell populations were counted automatically using Celigo. Each datapoint represents the mean of triplicate measurements ± s.d. The average number of all cells for each measurement varied from 13,525 ± 3,015 cells at 18 h to 35,715 cells ± 7,704 cells at 48 h post-infection. Experiments were performed once.

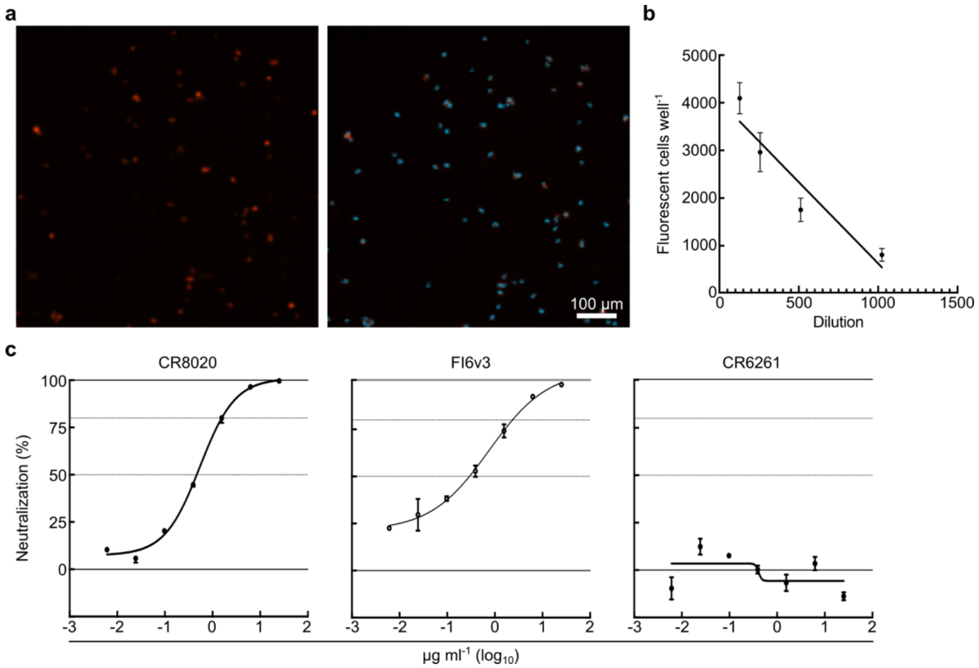

Supplementary Fig. 3. Neutralization assay using R3ΔPB1 influenza virus. **a,** R3ΔPB1 A/Singapore/INFIMH-16-0019/2016 (H3N2) virus-infected MDCK-SIAT1-PB1 cells at 18 hours post infection. Images representing single wells of 384-well plate are acquired using Target 1 protocol of Celigo. Fluorescent foci (left panel: red foci) are identified and counted automatically (right panel: foci with blue contour) using manufacturer software. **b,** Titration of R3ΔPB1 A/Singapore/INFIMH-16-0019/2016 (H3N2) virus. Linear range of fluorescence readings against virus dilutions is shown ($R^2 = 0.8519$). **c,** Representative neutralization profiles of three mAbs against R3ΔPB1 A/Singapore/INFIMH-16-0019/2016 (H3N2) virus. Normalized readings with standard deviations and fitted curves are shown for each mAb. The 50% (IC_{50}) and 80% (IC_{80}) inhibitory concentrations are obtained for each antibody from the fitted curve and shown with dotted lines. Each datapoint represents the mean of quadruplicate measurements ± s.d. Experments were performed at least ten times and representative data were shown.

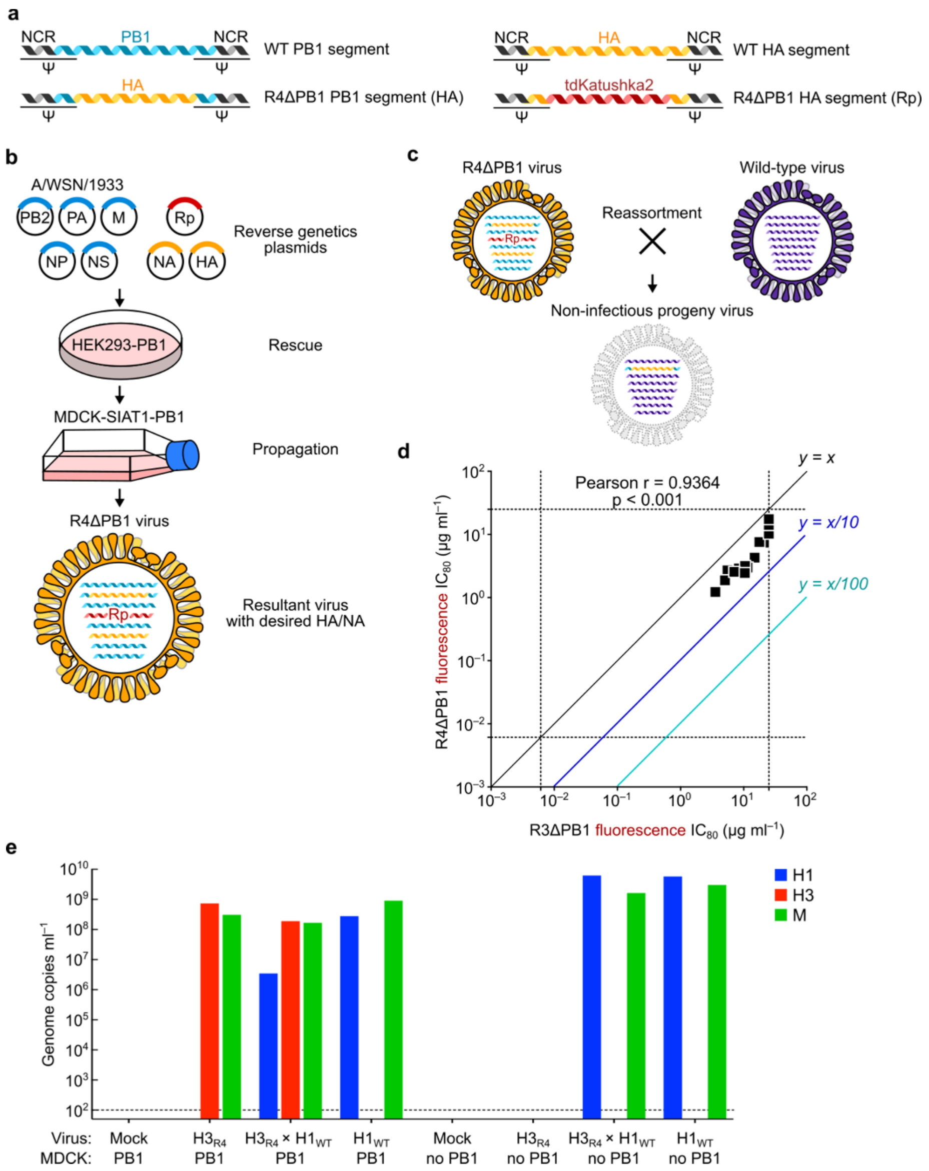

Supplementary Fig. 4. Generation of rewired replication-restricted reporter (R4) ΔPB1 influenza virus. **a,** Design of modified PB1 and HA segments used for R4ΔPB1 virus. PB1 segment contains PB1 packaging signals and HA ORF with mutated packaging signals. HA segment contains the HA packaging signals flanking the reporter ORF. **b,** R4ΔPB1 virus rescue and propagation. **c,** Non-viable reassortment between R4ΔPB1 and wild-type influenza viruses. Reassortant virus carrying the engineered PB1 segment encoding HA ORF and wild-type HA segment results in replication-deficient virus due to lack of PB1 gene. **d,** Correlation between neutralization titers of 15 mAbs against R3ΔPB1 and R4ΔPB1 viruses (A/Switzerland/9715293/2013). Each dot indicates titers (IC_{80} μg ml^{-1}) of a single mAb against R4ΔPB1 (y-axis) and R3ΔPB1 (x-axis) determined by fluorescent readout. Experiments were performed twice and representive data were shown. **e,** qRT-PCR analysis of influenza viruses to detect reassortment event. Forced reassortment experiment between R4ΔPB1 H3N2 (A/Switzerland/9715293/2013, $H3_{R4}$) and H1N1 (A/Solomon Islands/03/2006, $H1_{WT}$) was performed in MDCK-SIAT1 expressing PB1 cells followed by

propagation in parental MDCK-SIAT1 cells. MDCK SIAT1 expressing PB1 were infected with $H3_{R4}$, $H1_{WT}$ or 1:1 mixture of the two viruses. Due to the low titer of $H3_{R4}$ (6,270 $TCID_{50}$ ml^{-1}), the first passage was done at MOI of 0.2. Supernatants were harvested 48 hours post-infection. Viruses were initially passaged 3 times on MDCK-SIAT1 expressing PB1 to maximize the chances of reassortment events between $H3_{R4}$ and $H1_{WT}$ viruses, and then passaged 6 times on parental MDCK-SIAT1 cells (no PB1) to allow propagation of reassortant viruses (labeled as no PB1). One virus sample for each datapoint was analyzed. Experiments were performed once.

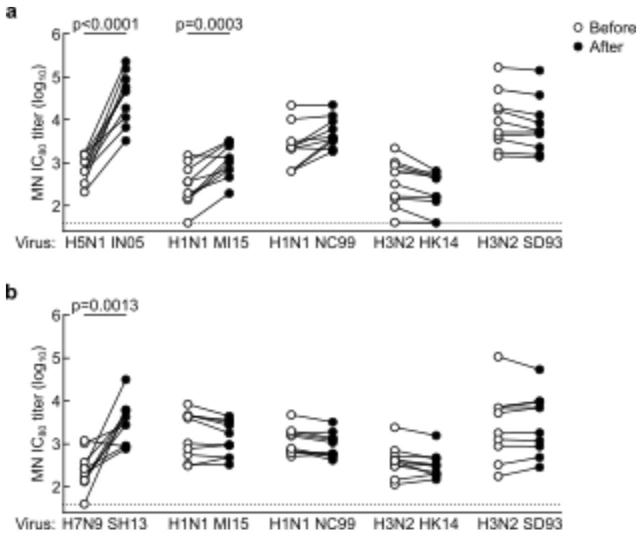

Supplementary Fig. 5. Assessment of breadth and depth of influenza responses in humans after H5 or H7 prime-boost regimens using R3 influenza viruses. **a,** Neutralization titers of the vaccine strain, H5N1 R3ΔHA A/Indonesia/05/2005, two H1N1 R3ΔPB1 (A/New Caledonia/20/1999 and A/Michigan/45/2014) and two H3N2 R3 R3ΔPB1 (A/Hong Kong/4801/2014 and A/Shangdong/9/1983) measured in ten human sera obtained at day 0, before priming with a DNA vaccine encoding H5 HA A/Indonesia/05/2005, and two weeks post-boost with a monovalent inactivated vaccine based on H5N1 A/Indonesia/05/2005 virus. **b,** Neutralization titers of the vaccine strain, H7N9 R3ΔHA A/Shanghai/02/2013, 2 H1N1 R3ΔPB1 (A/New Caledonia/20/1999 and A/Michigan/45/2014) and 2 H3N2 R3 R3ΔPB1 (A/ A/Hong Kong/4801/2014 and A/Shangdong/9/1983) measured in ten human sera obtained at day 0, before priming with a DNA vaccine encoding H7 HA A/Anhui/01/2013, and two weeks post-boost with a monovalent inactivated vaccine based on H7N9 A/Shanghai/02/2013 virus. Dotted lines indicate limit of detection. All *P* values were determined by using two-sided, paired Student's *t* tests in log measurements and groups with *P* values less than 0.005 are shown. Experiments were performed once.

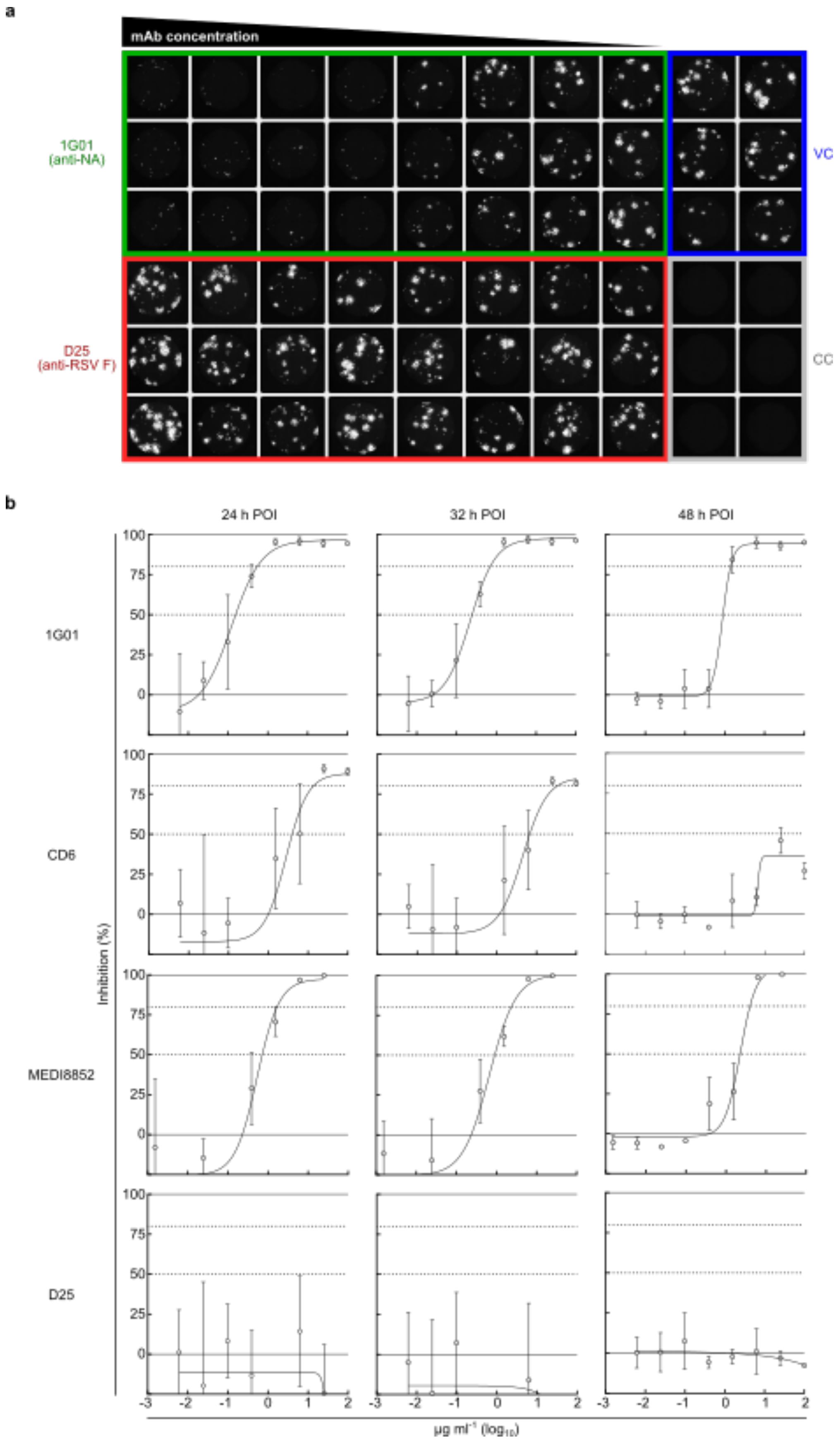
a
mAb concentration
1G01
(anti-NA)
VC
D25
(anti-RSV F)
CC
b
24 h POI
32 h POI
48 h POI
1G01
CD6
MEDI8852
D25
Inhibition (%)
µg ml⁻¹ (log10)

Supplementary Fig. 6. Plaque reduction assay using R3ΔPB1 A/California/07/2009 (H1N1) influenza virus. a, 96-well plate scan with virus-infected MDCK-SIAT1-PB1 cells at 24 hours post infection. Total area of fluorescent cells is identified and counted automatically using Confluency 1 protocol of Celigo. Wells containing cells pre-treated 4-fold dilutions of anti-NA 1G01 mAbs are highlighted in green, anti-RSV F D25 mAbs are highlighted in red. Control wells of virus alone (VC) are highlighted in blue and diluent alone (CC) wells are highlighted in gray. b, Normalized inhibition curves of two anti-NA mAbs, 1G01 and CD6, an anti-HA mAbs, MEDI8852, and anti-RSV F mAb, D25, at 24, 32 and 48 h post infection (POI). Each datapoint represents the mean of triplicate measurements ± s.d. Experiments were performed at least twice and representative data were shown.

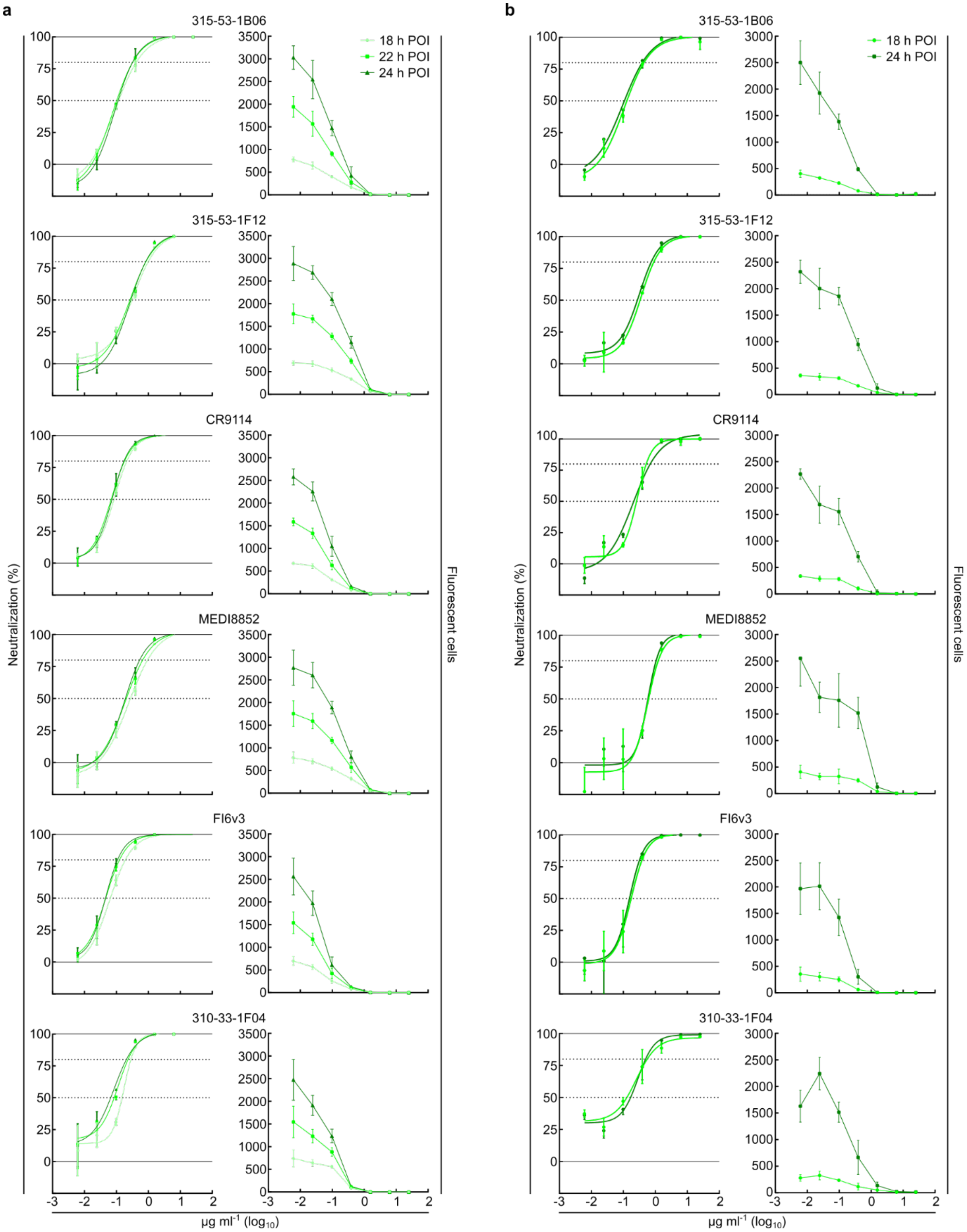

Supplementary Fig. 7. Neutralization curves of representative mAbs against R3ΔPB1 influenza viruses at different times post-infection. Neutralization curves were generated by using the infected cell counts at different time points. Overlay of neutralization curves (left panels) and raw fluorescent cell counts per well

(right panels) in the presence of six representative mAbs against R3ΔPB1 A/New Jersey/8/1976 (H1N1) at 18, 22 and 24 h post infection (POI) (**a**) and R3ΔPB1 A/California/07/2009 (H1N1) at 18 and 24 h POI (**b**). Each datapoint represents the mean of triplicate measurements ± s.d. Experiments were performed at least twice and representative data were shown.

Supplementary Table 1. Primers and probes used for qRT-PCR.

Name of primer/probe	Target	Sequence (5'–3')
NIID-H3 TMPrimer-F1	H3 HA	CTATTGGACAATAGTAAAACCGGGRGA
NIID-H3 TMPrimer-R1	H3 HA	GTCATTGGGRATGCTTCCATTTGG
NIID-H3 Probe1	H3 HA	(FAM)-AAGTAACCCCKAGGAGCAATTAG-(MGB)
MP-39-67For	Matrix	CCMAGGTCGAAACGTAYGTTCTCTCTATC
MP-183-153Rev	Matrix	TGACAGRATYGGTCTTGTCTTTAGCCAYTCCA
MP-96-75ProbeAs	Matrix	(FAM)-ATYTCGGCTTTGAGGGGGCCTG-(MGB)
IAVH1-5Fv2.2	H1 HA	AAAAAGCACACAAAATGCCATTAA
IAVH1-5Rv2.2	H1 HA	TCYAATTTGTTGAATTCTTTRCCCACA
IAVH1-5Pv2.2	H1 HA	(FAM)-YGGGATTACAAACAAGGTGAAYTCTGT-(MGB)

Supplementary Note 1

Protocol for
Production of replication-restricted reporter influenza viruses (R3ΔPB1)

Contents

A. Purpose
B. Procedure workflow
C. Procedure timeline
D. Experimental procedures

A. The purpose of this protocol is to define the methods for the following experiments:

1. Thawing and passaging of PB1-expressing cells (i.e., HEK-293 and MDCK-SIAT1).
2. Rescue a R3ΔPB1 influenza virus containing HA and NA genes from H1N1 and H3N2 influenza viruses, five internal genes (i.e., PB2, PA, NP, M and NS) from standard laboratory strains (e.g., A/WSN/1933 or A/Puerto Rico/8/1934) and a reporter gene, which will replace influenza PB1 coding sequence, using reverse genetics methods.
3. Propagation of R3ΔPB1 virus in MDCK-SIAT1 cells expressing influenza PB1 and prepare a 20 ml virus stock.

B. Procedure workflow:

1. Preparation of HEK-293 cells expressing PB1 for transfection.
2. Transfection of HEK-293 cells expressing PB1.
3. Propagation of rescued virus in PB1-expressing MDCK-SIAT1 cells for 4 passages.
4. Sequencing of HA, NA genes of R3ΔPB1 virus (recommended, protocol not provided here).

C. Detailed timeline:

**Time**	**Procedure**	**Note**
Week 1, Day 1	1.Thaw PB1 expressing HEK-293 and MDCK-SIAT1 cells<br>2.Solubilize and filter TPCK-treated trypsin powder	Recommend passaging HEK-293 cells once or twice before first transfection/infection
Week 1	Passage PB1-expressing cells	
Week 2, Day 1 (Monday)	Prepare PB1-expressing HEK-293 for transfection	
Week 2, Day 2 (Tuesday)	Transfect PB1-expressing HEK-293 cells	Cells should 40-60% confluent at the time of transfection. If cell confluency is higher than 60%,

		virus rescue efficiency will decrease
Week 2, Day 3 (Wednesday)	Observe transfected cells	Significant number of cells will express the reporter
Week 2, Day 4 (Thursday)	Observe transfected cells<br>Prepare PB1-expressing MDCK-SIAT1 for infection (Passage 2, 6-well plate, $3x10^5$ cells/well for 90-95% cell confluency at infection)	
Week 2, Day 5 (Friday)	1.Add trypsin to transfected cells 2-4h before harvest<br>2.Harvest virus (1st passage), aliquot and freeze<br>3.Infect PB1-expressing MDCK-SIAT1 cells	
Week 2, Day 6,7 (Saturday, Sunday) optional	Observe infected cells	
Week 3, Day 1	Harvest virus (2nd passage), aliquot and freeze<br>Prepare PB1 expressing MDCK-SIAT1 cells for passage 3 (T25, $8x10^5$ cells/flask for 90-95% cell confluency at infection)	If number of reporter-expressing cells and cytopathogenic effects (CPE) are not significant, consider:<br>1. Incubate the cells longer (up to 10 days post-infection) and observe them daily for reporter expression and CPE (add fresh TPCK-treated trypsin every 2-3 days)<br>2. Repeat transfection<br>3. Rescue with other HA, NA genes
Week 3, Day 2	Infect PB1-expressing MDCK-SIAT1 cells for passage 3	
Week 3, Day 3	Check reporter expression in infected cells	
Week 3, Day 4	Harvest virus (passage 3), aliquot, freeze	Harvest the flask infected with the highest virus dilution, which has 100% cells expressing the reporter and 70-90% CPE.<br>(use hemagglutinin assay or NA-Fluor to measure virus titer)
Week 3	Maintain cells	
Week 4, day 1	Prepare PB1 expressing MDCK-SIAT1 cells for passage 4 (T75, $3x10^6$ cells/flask for 90-95% cell confluency at infection)	

Week 4, day 2	Infect PB1-expressing MDCK-SIAT1 cells for passage 4	
Week 4, day 3	Check reporter expression in infected cells	
Week 4, day 4	Harvest virus (passage 4), aliquot, freeze	Harvest the flask infected with the highest virus dilution, which has 100% cells expressing the reporter and 70-90% CPE. (use hemagglutinin assay or NA-Fluor to measure virus titer)
Week 4	Continue to maintain the cells	
Week 5, day 1	Prepare PB1 expressing MDCK-SIAT1 cells passage 5 (T175, 6×10^6 cells/flask for 90-95% cell confluency at infection)	
Week 5, day 2	Infect PB1-expressing MDCK-SIAT1 cells for passage 5	
Week 5, day 3	Check reporter expression in infected cells	
Week 5, day 4	Harvest virus (passage 5), aliquot, freeze	Harvest the flask infected with the highest virus dilution, which has 100% cells expressing the reporter and 70-90% CPE. (use hemagglutinin assay or NA-Fluor to measure virus titer)

Notes:
To prevent virus cross-contamination, handle a single virus in the tissue culture cabinet at a time. Disinfect well the cabinet (spray with 70% ethanol, turn on UV light for at least 15-30 minutes) and other materials (e.g., pipette aids, pipette, tip boxes, bottles, etc.), change gloves before another virus is handled in the same cabinet. If multiple viruses are passaged in the same day in the same hood, consider passaging viruses with different HA and NA subtypes. Cross-contamination with viruses of different subtypes are easier to detect by sequencing. Cross-contamination of viruses with similar HA, NA genes is difficult to detect even by standard sequencing procedure.

D. Experimental Procedures

D.1. Rescue of replication-restricted reporter influenza viruses

D.1.1. Thawing PB1-expressing cells for initial cultivation and subsequent passage

Materials and Supplies:

(a) Frozen PB1-expressing cell lines (i.e., HEK-293 and MDCK-SIAT1)
(b) Complete DMEM prepared with 445 ml DMEM (High-Glucose, L-Glutamine, Pyruvate: Thermo Fisher Cat # 11995073), 5 ml of 100X Penicillin/Streptomycin (VWR Cat # 16777-164) and 50 ml of heat inactivated fetal Bovine Serum (Benchmark FBS, Gemini Bio-Products, Cat # 100-106)

(c) Conical 15-ml tubes
(d) 10- and 25-ml Pipettes
(e) T75 tissue culture treated, sterile (VWR Cat # 82050-856)
(f) 70% Ethanol.

Equipment:
(a) Water bath
(b) CO_2 incubator
(c) Refrigerator
(d) Biosafety cabinet (BSC)
(e) Centrifuge
(f) Portable pipet aid.

Methods:
(a) Spray the surface of material and supplies with 70% ethanol and bring them into BSC.
(b) Turn on water bath and set temperature at 37°C.
(c) Bring cells from liquid nitrogen.
(d) Place cells in water bath until thawed.
(e) Spray the surface of vial with 70% ethanol.
(f) Transfer the contents of vial to 15 ml conical tube.
(g) Add ~10 ml of complete DMEM into tube and incubate for 5-10 minutes.
(h) Centrifuge tube at 1,000 rpm/200*g* for 5 minutes.
(i) Discard supernatant.
(j) Re-suspend cells in appropriate volume of complete DMEM (e.g., 15 ml for T75 flask).
(k) Seed cell suspension into T75 tissue culture flasks.
(l) Place flasks or plates in CO_2 incubator.
(m) Incubate cells at 37°C, 5% CO_2 and humidity.
(n) Store remaining medium in refrigerator at 4°C.

D.1.2. Change the medium of PB1-expressing cells one day post-thawing

Materials and Supplies:
a) Cultured PB1-expressing cells (as described in “Thawing PB1-expressing cells for initial cultivation”)
b) Complete DMEM (as described in “Thawing PB1-expressing cells for initial cultivation”)
c) 10- and 25-ml pipettes
d) 70% Ethanol.

Equipment:
a) As described in “Thawing PB1-expressing cells for initial cultivation and subsequent passage”.

Methods:
a) Spray the surface of material and supplies with 70% ethanol and bring them into BSC.
b) Warm up Complete DMEM medium at 37°C using the water bath.

c) Take culture flasks or plates out from CO_2 incubator.
d) Place flask under the microscope.
e) Observe the cells. Most of the cells are adherent to the flask one day post-thawing.
f) Bring the flask into the BSC.
g) Remove the supernatant gently without disturbing the cell monolayer.
h) Add appropriate volume of complete DMEM (e.g., 20 ml per T75 flask).
i) Observe cells at the microscope.
j) Record the approximate cell confluence as a percent of surface area.
k) Return culture flasks to CO_2 incubator.
l) Incubate cells at 37°C, 5% CO_2 and humidity until they reach 90-95% cell confluency as a percent of surface area.

D.1.3. Passage of PB1-expressing cells

Materials and Supplies:

a) Cultured PB1-expressing cells (as described in “Thawing PB1-expressing cells for initial cultivation”)
b) Complete DMEM (as described in “Thawing PB1-expressing cells for initial cultivation”)
   Note: after 2-3 passages post-thawing, complete DMEM medium for PB1-expressing MDCK-SIAT1 cells is supplemented with Puromycin (0.25 µg/ml, Thermo Fisher, Cat # A1113803) and Geneticin (1mg/ml, Thermo Fisher Cat #10131027).
c) 10- and 25-ml pipettes
d) 70% Ethanol
e) Trypsin, 0.25% 1X Solution, with 2.5 g Porcine Trypsin (1:250/L) in HBSS with 0.2 g/L EDTA, without Calcium or Magnesium (VWR Cat # 16777-166)
f) PBS pH 7.4, sterile (Quality Biological Cat # 114-058-101).

Equipment:

a) As described in “Thawing PB1-expressing cells for initial cultivation and subsequent passage”.

Methods:

a) Spray the surface of material and supplies with 70% ethanol and bring them into BSC.
b) Warm up Complete DMEM medium at 37°C using the water bath.
c) Take culture flasks out from CO2 incubator.
d) Place flask under the microscope.
e) Observe the cells.
f) Record the approximate cell confluence as a percent of surface area.
   Note: Choose the flask with 80-95% cell confluency
g) Bring the flask into the BSC.
h) Remove the supernatant gently without disturbing the cell monolayer.
i) Wash cells with the appropriate volume of PBS (10 ml per T75 flask).
j) Rinse cells by tilting flasks and remove PBS.

k) Repeat the washing steps only when PB1-expressing MDCK-SIAT1 cells are passaged.
l) Add 3 ml of Trypsin per T75 flask.
m) Ensure cells are covered with Trypsin by tilting flasks.
n) Place flasks in CO_2 incubator and incubate (3-5 minutes for HEK-293 cells or 15-20 minutes for MDCK-SIAT1 cells).
o) Remove flasks from CO_2 incubator and bring them into the BSC.
p) Detach cells by tapping flasks.
q) Add up to 10 ml complete DMEM and re-suspend cells by pipetting.
r) Pool cell suspension from flasks into one flask if trypsinizing multiple flasks.
s) Determine the dilution of cell suspension depending upon when the confluent cell cultures are needed.
   Note: We passage the PB1-expressing cells every 2-3 days. 1 ml out of the 10ml cell suspension prepared from a single flask with cells at 90-95% cell confluency can be transferred to the new flask, which will be confluent 48 h later.
t) Distribute cell suspension into culture flasks.

Note: We maintain both cell lines up to 20 passages (each passage is 2-3 days)
Note: We freeze PB1-expressing cells in complete Media (90%) and DMSO (10%, molecular biology grade, Sigma Cat D8418-50ML) following standard procedure (3-10 million cells per ml in a single cryovial).

D.1.4. Plating PB1-expressing HEK-293 cells for transfection

Materials and Supplies:

a) Cultured HEK-293 PB1-expressing cells (as described in “Thawing PB1-expressing cells for initial cultivation”)
b) Complete DMEM (as described in “Thawing PB1-expressing cells for initial cultivation”)
c) Trypsin, 0.25% 1X Solution, with 2.5 g Porcine Trypsin (1:250/L) in HBSS with 0.2 g/L EDTA, without Calcium or Magnesium (VWR Cat# 16777-166)
d) PBS pH 7.4, sterile (Quality Biological Cat# 114-058-101)
e) Trypan blue (Bio Rad, Cat 1450021)
f) 6-well Plate: Corning BioCoat™ Poly-D-Lysine Multiwell Plates (354413) (Fisher Scientific Cat # 08-774-268)
   Note: Use 6 well plates coated with Poly-D-Lysine to improve HEK-293 cell adhesion
g) Cell counting supplies (hemocytometer, slides)
h) 10- and 25-ml pipettes
i) Pipette tips (200)
j) 70% Ethanol.

Equipment:

a) Biosafety cabinet (BSC)
b) Portable Pipet-Aid
c) Pipettes (P200)

d) Microscope
e) CO_2 incubator
f) Refrigerator
g) Water bath set at 37°C
h) Cell counter (optional).

Methods:

a) Spray the surface of material and supplies with 70% ethanol and bring them into BSC.
b) Warm up Complete DMEM medium at 37°C using the water bath.
c) Take culture flasks out from CO_2 incubator.
d) Place flask under the microscope.
e) Observe the cells.
f) Record the approximate cell confluence as a percent of surface area.
   Note: Choose the flask with 80-95% cell confluency.
g) Bring the flask into the BSC.
h) Remove the supernatant gently without disturbing the cell monolayer.
i) Wash cells with the appropriate volume of PBS (10 ml per T75 flask).
j) Rinse cells by tilting flasks and remove PBS.
k) Add 3 ml of Trypsin per T75 flask.
l) Ensure cells are covered with Trypsin by tilting flasks.
m) Place flasks in CO_2 incubator and incubate for 3-5 minutes.
n) Remove flasks from CO_2 incubator and bring them into the BSC.
o) Detach cells by tapping flasks.
p) Add up to 10 ml complete DMEM and re-suspend cells by pipetting.
q) Count cells (10-20 million cells from T75 flask) and dilute them to 0.2-0.3 million. cells per ml if cells are prepared 20h before transfection. Add 2 ml per well of 6-well plate.
   Note: Cell confluency at the time of transfection should be 40-60%. It is recommended to prepare cells at 2-3 different dilutions and transfection is performed on wells with the 40-60% cell confluency.
r) Passage the remaining cells as described in “PB1-expressing cells passage” for future usage.
s) Return the 6-well plate and flask with cells in the incubator and incubate until they reach 90-95% confluency.

D.1.5. Transfection of PB1-expressing HEK-293 cells and addition of trypsin

Materials and Supplies:

a) PB1-expressing HEK-293 cells in 6-well plate treated with Poly-D-Lysine as described in “Plating PB1-expressing HEK-293 cells for transfection”
b) Plasmid DNA (HA, NA, five internal genes-PB2, PA, NP, M and NS of A/WSN/1933, one reporter, PB1packiging TdKatushka2-NLS, and hTMPRSS2)
c) Lipofectamine 3000 (Thermo Fisher, Cat # L3000001)
d) Complete DMEM (as described in “Thawing PB1-expressing cells for initial cultivation”)

e) Trypsin, 0.25% 1X Solution, with 2.5 g Porcine Trypsin (1:250/L) in HBSS with 0.2 g/L EDTA, without Calcium or Magnesium (VWR Cat# 16777-166)
f) PBS pH 7.4, sterile (Quality Biological Cat# 114-058-101)
g) Opti-MEM I Reduced-Serum Medium without Phenol-Red (Thermo Fisher Cat # 11058021)
h) TPCK-treated trypsin (Sigma, Cat# T1426-50MG). 50 mg powder is mixed with 50ml sterile water (Thermo Fisher, Cat# 4387937) at 1mg/ml, solution is filtered with 50-ml disposable filter units and 200μl aliquots are stored at -20°C for 1-2 years.
i) 10- and 25-ml pipettes
j) Pipette tips (10, 200, 1000)
k) 2.0 ml Cryotubes (Sarstedt, Fisher Scientific Cat # 50-809-242)
l) Pipette (P2, P20, P200, P1000)
m) 70% Ethanol
n) Disposable 50-ml filter units (Corning, Cat # 430320).

Equipment:
a) Biosafety cabinet
b) Portable Pipet-Aid
c) Pipettes (P2, P20, P200, P1000)
d) Pipette tips (20 μl, 200 μl, 1000 μl)
e) Microscope
f) CO_2 incubator
g) Refrigerator
h) Water bath set at 37°C
i) Vortex
j) Freezer -80°C
k) High Speed Centrifuge.

Methods:
a) Spray the surface of material and supplies with 70% ethanol and bring them into the biosafety cabinet (BSC).
b) Warm up Opti-MEM medium at 37°C using the water bath.
c) Take 6-well plate out from CO_2 incubator.
d) Place the plate under the microscope.
e) Check the cells seeded on 6-well plates under microscope.
f) Record the approximate cell confluence as a percent of surface area.
Note: Choose the wells with 40-60% cell confluency.
g) Bring the plate into the BSC.
h) Remove the supernatant gently without disturbing the cell monolayer.
i) Wash cells twice with 2 ml PBS.
j) Remove PBS.
k) Add 1 ml of Opti-MEM.
l) Prepare the Opti-MEM-plasmid DNA-P3000 mix in this: Add to a 2-ml cryotube for a single 6-well: 125 μl Opti-MEM, 1μg of each plasmid encoding influenza segment (8

plasmids) and 0.3 µg of pTMPRSS2 plasmid and 2 µl/µg of DNA of P3000 reagent (16.6 µl)

Note: Replication-restricted reporter ΔPB1 virus can be rescued by transfecting HEK-293 cells with a mixture of plasmids, which includes the pol-II-driven expression plasmid encoding PB1 of A/WSN/1933.

	**P3000 mix (per well)**
Opti-MEM	125 µl
P3000	2 µl per µg of DNA
Plasmid DNA: PB2	1 µg
Plasmid DNA: PB1-reporter	1 µg
Plasmid DNA: (POL-II only) PB1	1 µg (only for HEK-293)
Plasmid DNA: PA	1 µg
Plasmid DNA: NP	1 µg
Plasmid DNA: M	1 µg
Plasmid DNA: NS	1 µg
Plasmid DNA: HA	1 µg
Plasmid DNA:NA	1 µg
Plasmid DNA: TMPRSS2	0.3 µg

m) Vortex the tube with Lipofectamine 3000 reagent.
n) Prepare the Opti-MEM-L3000 mix: Add to a 2-ml cryotube for a single 6-well: 125 µl Opti-MEM and 10 µl Lipofectamine 3000. Pipette up and down, vortex.

	**L3000 mix (per well)**
Opti-MEM	125 µl
L3000	10 µl

o) Transfer 135 µl of Opti-MEM-L3000 mix to Opti-MEM-plasmidDNA-P3000 mix. Mix well.
p) Incubate 10-15 minutes at room temperature.
q) Transfer the DNA-transfection reagent mix (285 µl) to a single well by pipetting (dropwise).
r) Transfer the plate to CO_2 incubator.
s) Incubate cells at 37°C, 5% CO_2 and humidity for 3 days.
t) You can monitor and record reporter expression daily.
u) Three days post transfection, take 6-well plate out from CO_2 incubator.
v) Place the plate under the microscope.
w) Observe the cells.
x) Record the approximate cell confluence as a percent of surface area and the percentage of cells expressing the reporter.
Note: 80-100% cells should express the reporter at this time point
y) Bring the plate to BSC.
z) Add TPCK-treated trypsin at 0.5-1 µg/ml to each well with transfected cells
Note: TPCK-treated trypsin is stored at 1 mg/ml (2000-1000X). Consider diluting TPCK-treated trypsin in this way: 130 µl of TPCK-treated trypsin 1 mg/ml to 10 ml

Opti-MEM and transfer 100µl of diluted TPCK-treated trypsin to a single well containing 1,300 µl medium for 1 µg/ml TPCK-treated trypsin

aa) Return culture plate to CO_2 incubator.

bb) Incubate cells for 2-4 hours at 37ºC, 5% CO_2 and humidity.
Note: TPCK-treated trypsin is required for HA cleavage and its activity is redundant with TMPRSS2. If HA cleavage is not cleaved efficiently, the virus is not infectious. However, TPCK-treated trypsin is toxic for HEK-293 cells and cells will detach quickly after trypsin addition. Thus, optimal incubations with TPCK-treated trypsin should not be longer than 2-4 hours.

cc) Remove transfected cells from CO_2 incubator and bring them into BSC.

dd) Remove the plates from CO_2 incubator. Observe the cells under microscope, and record the confluency and conditions of transfected cells

ee) Bring the plate into BSC.

ff) Transfer cell suspension to a 2-ml cryotube and centrifuge for 5 minutes at 200-400*g*

gg) Bring the tube into BSC.

hh) Aliquot the supernatant to pre-labeled 2-ml cryotubes and transfer the aliquots to -80°C.

D.2. Plating PB1-expressing MDCK-SIAT1 cells for infection (Passage 2 6-well plate)

Materials and Supplies:

a) PB1-expressing MDCK-SIAT cells plated in T75 flask as described in “Thawing PB1-expressing cells for initial cultivation” or “PB1-expressing cells passage”
b) 6-well plate (Falcon Polystyrene Microplates, Fisher Scientific Cat # 08-772-1B)
Note: Please use plates and flasks with standard tissue culture treated surfaces to grow MDCK-SIAT1 cell lines; do NOT use plates or flasks with poly-D-lysine treated surfaces to grow MDCK-SIAT1 cell lines
c) Complete DMEM (as described in “Thawing PB1-expressing cells for initial cultivation”)
Note: Complete DMEM does not contain Puromycin or Geneticin.
d) Trypsin, 0.25% 1X Solution, with 2.5 g Porcine Trypsin (1:250/L) in HBSS with 0.2 g/L EDTA, without Calcium or Magnesium (VWR Cat # 16777-166)
e) PBS pH 7.4, sterile (Quality Biological Cat # 114-058-101)
f) 10- and 25-ml pipettes
g) Pipette tips (10, 200, 1000)
h) 70% Ethanol
i) 15-ml conical tube
j) Cell counting consumables (trypan blue, hemocytometer).

Equipment:

a) Biosafety cabinet (BSC)
b) Microscope
c) Portable Pipet-Aid
d) Pipettes (P2, P20, P200, P1000)
e) Cell counter (optional)

f) CO_2 incubator
g) Refrigerator
h) Water bath set at 37°C.

Methods:

a) Spray the surface of material and supplies with 70% ethanol and bring them into BSC.
b) Warm up Complete DMEM medium at 37°C using the water bath.
c) Take culture flasks out from 37°C CO_2 incubator.
d) Place flask under the microscope.
e) Observe the cells.
f) Record the approximate cell confluence as a percent of surface area.
   Note: Choose the flask with 80-95% cell confluency
g) Bring the flask into the BSC.
h) Remove the supernatant gently without disturbing the cell monolayer.
i) Wash cells with the appropriate volume of PBS (2-3 ml per T75 flask).
j) Rinse cells by tilting flasks and remove PBS.
k) Repeat the wash step.
l) Add 3 ml of trypsin per T75 flask.
m) Ensure cells are covered with Trypsin by tilting flasks.
n) Place flasks in CO_2 incubator and incubate for 15-20 minutes.
o) Tap the flask every 5-10 minutes.
p) Remove flask from CO_2 incubator and bring them into the BSC.
q) Detach cells by tapping flasks.
r) Add up to 10 ml complete DMEM and re-suspend cells by pipetting.
s) Count cells (10-20 millions cells from T75 flask) and dilute them to 0.15 million (1.5×10^5) cells per ml if cells are plated 20 h before transfection.
t) Add 2 ml per well of 6-well plate (one plate per virus).
u) Passage the remaining cells as described in “PB1-expressing cells passage” for future usage.
v) Return the 6-well plate and flask with cells in the incubator and incubate until 90-95% cell confluency.

D.2.1. Infecting PB1-expressing MDCK-SIAT1 cells (Passage 2 6-well plate)

Materials and Supplies:

a) PB1-expressing MDCK-SIAT cells as described in “Plating PB1-expressing MDCK-SIAT1 cells for infection (Passage 2 6-well plate)”
o) Opti-MEM I Reduced-Serum Medium without Phenol-Red (Thermo Fisher Cat # 11058021)
b) PBS pH 7.4, sterile (Quality Biological Cat# 114-058-101)
c) TPCK-treated trypsin (Sigma, Cat# T1426-50MG). Powder is mixed with distilled water at 1mg/ml, solution is filtered and 200 μl aliquots are stored at -20°C for 1-2 years.
d) 10- and 25-ml pipettes
e) Pipette tips (10, 200, 1000)

f) 70% Ethanol
g) 15-ml conical tube
h) 2-ml Cryotube (Sarstedt, Fisher Scientific Cat # 50-809-242)
i) 10% bleach freshly prepared from concentrated solution.

Equipment:

a) Biosafety cabinet (BSC)
b) Microscope
c) Portable Pipet-Aid
d) CO_2 incubator at 37ºC
e) Refrigerator
f) Water bath set at 37°C
g) Label printer
h) -80ºC Freezer
i) Centrifuge.

Methods:

a) Spray the surface of material and supplies with 70% ethanol and bring them into BSC.
b) Warm up Opti-MEM medium at 37°C using the water bath.
c) Take the plate with PB1-expressing cells out from CO_2 incubator.
d) Place plate under the microscope.
e) Observe the cells.
f) Record the approximate cell confluence as a percent of surface area.
   Note: Cell confluency should be 80-95%.
g) Bring the 6-well plate into the BSC.
h) Remove the supernatant gently without disturbing the cell monolayer.
i) Wash cells twice with the appropriate volume of PBS (2 ml per T75 flask).
j) Keep the cells in PBS in the incubator until virus dilutions are ready (no more than 5-10 minutes).
k) Bring the virus aliquot from -80°C.
l) Thaw the virus in the water bath at 37°C.
m) Prepare 3-4 10-fold dilutions in Opti-MEM with (optional) TPCK-treated trypsin at 1 µg/ml.
n) Aspirate PBS from each well with PB1-expressing MDCK-SIAT1 cells.
o) Add 0.3 ml virus dilutions (first dilution is “neat”) to each well.
p) Ensure cells are covered with solution by tilting flasks.
q) Incubate the plate for 1 h at 37°C..
r) Tilt the plate every 10-15 minutes to ensure cells are covered with solution.
s) Bring the plate into the BSC.
t) Remove virus inoculum.
   Note: consider leaving the inoculum for neat if virus rescue can be low
u) Do not wash the cells to improve rescue efficiency.
v) Add 1.5 ml Opti-MEM with 1 µg/ml TPCK-treated trypsin to each well.
w) Return the 6-well plate in the CO2 incubator at 37°C.

x) Monitor the cells and record daily for reporter expression and cytopathogenic effects.
y) Prepare labels for virus aliquots (label should contain full virus name, passage number, date, volume, other relevant information).
z) Harvest at 72 h post infection the well infected with the highest dilution, which has 100% reporter expressing cells, significant cytopathogenic effects (70-90% cells are detached or have altered morphology) and 30-10% cells are still attached and have normal morphology. Virus from wells or flasks with 100% reporter expressing cells and 100% CPE is considered overgrown and not harvested.
Note: (recommended) Hemagglutination assay or neuraminidase assay (NA-Fluor, Thermo Fisher Cat # 4457091) can be used to measure virus titer. Of note, recent H3N2 seasonal strains do not agglutinate red blood cells efficiently
Note: If the well infected with neat (undiluted virus) does not have 80-100% cells expressing the reporter consider incubating longer (up to 10 days) and add fresh TPCK-treated trypsin (0.1-0.2 µg per well every 48h). Continue to monitor cells daily. Consider harvesting aliquots only when at least 50% cells express the reporter. If less than 10% cells express the reporter 10 days post infection, consider repeating the transfection or rescue a virus with different HA and NA genes.
aa) Transfer cell suspension to a conical tube (e.g., 15- or 50-ml) centrifuge for 5-10 minutes at 200-400*g*.
bb) Bring the tube into BSC.
cc) Aliquot the supernatant to pre-labeled 2-ml cryotubes and transfer the aliquots to -80°C for later usage.
dd) Inactivate the virus from wells which were not harvested by adding one volume of 10% bleach and incubate for at least 15-30 minutes.
ee) Discard inactivated viruses following internal safety procedures.

D.2.2. Plating PB1-expressing MDCK-SIAT1 cells for infection (Passage 3 T25 flask)

Materials and Supplies:

a) PB1-expressing MDCK-SIAT cells as described in “as described in “Thawing PB1-expressing cells for initial cultivation” or “PB1-expressing cells passage”
b) T25 flask (TC treated, e.g., Fisher Scientific Cat # 82051-074)
Note: Please use plates and flasks with standard tissue culture treated surfaces to grow MDCK-SIAT1 cell lines; do NOT use plates or flask with poly-D-lysine treated surfaces to grow MDCK-SIAT1 cell lines
c) All the supplies described in “Plating PB1-expressing MDCK-SIAT1 cells for infection (Passage 2 6-well plate)”.

Equipment:

a) Same as described in “Plating PB1-expressing MDCK-SIAT1 cells for infection (Passage 2 6-well plate)”.

Procedure:

a) Same as described in “Plating PB1-expressing MDCK-SIAT1 cells for infection (Passage 2 6-well plate)”
b) Count cells (10-20 million cells from T75 flask) and dilute them to 0.8 million (8×10^5) cells per flask if cells are plated 20 h before infection. Cells should have 80-95% confluency at the time of infection. Add 5 ml complete medium per flask (4-6 flasks for each virus).

D.2.3. Infecting PB1-expressing MDCK-SIAT1 cells (Passage 3 T25 flask)

Materials and Supplies:

a) PB1-expressing MDCK-SIAT cells as described in “Plating PB1-expressing MDCK-SIAT1 cells for infection (Passage 3 T25 flask)”
b) Same as described in “Infecting PB1-expressing MDCK-SIAT1 cells (Passage 2 6-well plate)”.

Equipment:

a) Same as described in “Infecting PB1-expressing MDCK-SIAT1 cells (Passage 2 6-well plate)”.

Procedure:

a) Largely as described in “Infecting PB1-expressing MDCK-SIAT1 cells (Passage 2 6-well plate)” with the following modifications:
b) Prepare 4-6 10-fold dilutions in Opti-MEM with (optional) TPCK-treated trypsin at 1 μg/ml.
c) Add 0.9 ml virus dilution to each flask.
d) Gently tilt the plate every 10-15 minutes to ensure cells are covered with solution.
e) After one-hour incubation, remove inoculum.
f) (optional) Wash each flask with 5 ml PBS.
g) Harvest at 48 h post infection the flask infected with the highest dilution, which has 100% reporter expressing cells, significant cytopathogenic effects (70-90% cells are detached or have altered morphology) and 30-10% cell are still attached and have normal morphology. Virus from wells or flasks with 100% reporter expressing cells and 100% CPE is considered overgrown and is not harvested.
   Note: (recommended) Hemagglutination assay or neuraminidase assay (NA-Fluor, Thermo Fisher Cat # 4457091) can be used to measure virus titer. Of note, recent H3N2 seasonal strains do not agglutinate efficiently red blood cells
h) Prepare aliquots in pre-labeled 2-ml cryotubes and transfer to -80°C.
i) Inactivate the virus from flasks which were not harvested by adding one volume of 10% bleach and incubate for at least 15-30 minutes.
j) Discard inactivated viruses following internal safety procedures.

D.2.4. Plating PB1-expressing MDCK-SIAT1 cells for infection (Passage 4 T75 flask)

Materials and Supplies:

a) PB1-expressing MDCK-SIAT cells as described in “as described in “Thawing PB1-expressing cells for initial cultivation” or “PB1-expressing cells passage”

b) T75 flask (TC treated, e.g., Fisher Scientific Cat # 07-000-229)
   Note: Please use plates and flasks with standard tissue culture treated surfaces to grow MDCK-SIAT1 cell lines; do NOT use plates or flask with poly-D-lysine treated surfaces to grow MDCK-SIAT1 cell lines
c) All the supplies described in “Plating PB1-expressing MDCK-SIAT1 cells for infection (Passage 2 6-well plate)”.

Equipment:

b) Same as described in “Plating PB1-expressing MDCK-SIAT1 cells for infection (Passage 2 6-well plate)”.

Procedure:

c) Same as described in “Plating PB1-expressing MDCK-SIAT1 cells for infection (Passage 2 6-well plate)”
d) Count cells (10-20 million cells from T75 flask) and dilute them to 3×10^6 cells per flask if cells are plated 20 h before infection. Cells should have 80-95% confluency at the time of infection. Add 15 ml complete medium per flask (4-6 flasks for each virus).

D.2.5. Infecting PB1-expressing MDCK-SIAT1 cells (Passage 4 T75 flask)

Materials and Supplies:

a) PB1-expressing MDCK-SIAT cells as described in “Plating PB1-expressing MDCK-SIAT1 cells for infection (Passage 4 T75 flask)”
b) Same as described in “Infecting PB1-expressing MDCK-SIAT1 cells (Passage 2 6-well plate)”.

Equipment:

a) Same as described in “Infecting PB1-expressing MDCK-SIAT1 cells (Passage 2 6-well plate)”.

Procedure:

a) Largely as described in “Infecting PB1-expressing MDCK-SIAT1 cells (Passage 2 6-well plate)” with the following modifications:
b) Prepare 4-6 4/10-fold dilutions in conical tubes with Opti-MEM with (optional) TPCK-treated trypsin at 1 µg/ml.
   Note: Use lower dilution factor for viruses which do not grow well.
c) Add 3 ml virus dilution to each flask.
d) Gently tilt the plate every 10-15 minutes to ensure cells are covered with solution.
e) After one-hour incubation, remove inoculum.
f) Wash each flask with 10 ml PBS.
g) Harvest at 48 h post infection the flask infected with the highest dilution, which has 100% reporter expressing cells, significant cytopathogenic effects (70-90% cells are detached or have altered morphology) and 30-10% cell are still attached and have normal morphology. Virus from wells or flasks with 100% reporter expressing cells and 100% CPE is considered overgrown and is not harvested.

Note: (recommended) Hemagglutination assay or Neuraminidase assay (NA-Fluor, Thermo Fisher Cat # 4457091) can be used to measure virus titer. Of note, recent H3N2 seasonal strains do not agglutinate efficiently red blood cells

h) Prepare aliquots in pre-labeled 2-ml cryotubes and transfer to -80°C.
i) Inactivate the virus from flasks which were not harvested by adding one volume of 10% bleach and incubate for at least 15-30 minutes.
j) Discard inactivated viruses following internal safety procedures.

D.2.6. Plating PB1-expressing MDCK-SIAT1 cells for infection (Passage 5 T175 flask)

Materials and Supplies:

a) PB1-expressing MDCK-SIAT cells as described in "as described in "Thawing PB1-expressing cells for initial cultivation" or "PB1-expressing cells passage"
b) T175 flask (TC treated, e.g., Fisher Scientific Cat # 07-000-384)
   Note: Please use plates and flasks with standard tissue culture treated surfaces to grow MDCK-SIAT1 cell lines; do NOT use plates or flask with poly-D-lysine treated surfaces to grow MDCK-SIAT1 cell lines
c) All the supplies described in "Plating PB1-expressing MDCK-SIAT1 cells for infection (Passage 2 6-well plate)".

Equipment:

a) Same as described in "Plating PB1-expressing MDCK-SIAT1 cells for infection (Passage 2 6-well plate)".

Procedure:

a) Same as described in "Plating PB1-expressing MDCK-SIAT1 cells for infection (Passage 2 6-well plate)".
b) Count cells (10-20 million cells from T75 flask) and dilute them to 6-7 $\times$ 10^6 cells per flask if cells are plated 20 h before infection. Cells should have 80-95% confluency at the time of infection. Add 27 ml complete medium per flask (4-6 flasks for each virus).

D.2.7. Infecting PB1-expressing MDCK-SIAT1 cells (Passage 5 T175 flask)

Materials and Supplies:

a) PB1-expressing MDCK-SIAT cells as described in "Plating PB1-expressing MDCK-SIAT1 cells for infection (Passage 5 T175 flask)"
b) Same as described in "Infecting PB1-expressing MDCK-SIAT1 cells (Passage 2 6-well plate)".

Equipment:

a) Same as described in "Infecting PB1-expressing MDCK-SIAT1 cells (Passage 2 6-well plate)".

Procedure:

a) Largely as described in "Infecting PB1-expressing MDCK-SIAT1 cells (Passage 2 6-well plate)" with the following modifications:

b) Prepare 4-6 3/6-fold dilutions in conical tubes with Opti-MEM with (optional) TPCK-treated trypsin at 1 μg/ml.
   Note: Use lower dilution factor for viruses which do not grow well.
c) Add 5 ml virus dilution to each flask.
d) Gently tilt the plate every 10-15 minutes to ensure cells are covered with solution.
e) After one-hour incubation, remove inoculum.
f) Wash each flask with 15 ml PBS.
g) Harvest at 48 h post infection the flask infected with the highest dilution, which has 100% reporter expressing cells, significant cytopathogenic effects (70-90% cells are detached or have altered morphology) and 30-10% cell are still attached and have normal morphology. Virus from wells or flasks with 100% reporter expressing cells and 100% CPE is considered overgrown and is not harvested.
   Note: (recommended) Hemagglutination assay or neuraminidase assay (NA-Fluor, Thermo Fisher Cat # 4457091) can be used to measure virus titer. Of note, recent H3N2 seasonal strains do not agglutinate efficiently red blood cells.
h) Prepare 0.1-0.5 ml aliquots in pre-labeled 2-ml cryotubes and transfer to -80°C.
i) Inactivate the virus from flasks which were not harvested by adding one volume of 10% bleach and incubate for at least 15-30 minutes.
j) Discard inactivated viruses following internal safety procedures.

Supplementary Note 2

Protocol for
Influenza neutralization assay in 384-well plate format using R3ΔPB1 influenza viruses

Contents

A. Purpose
B. Procedure timeline
C. Experimental procedures

A. The purpose of this protocol is to define the methods for the following experiments:

1. Treatment of serum samples with Receptor destroying enzyme (RDE)
2. Titration of R3ΔPB1 influenza viruses in 384-well plates using image-based plate reader
3. Influenza neutralization assay using R3ΔPB1 in 384-well plates

B. Procedure timeline:

Day 0: RDE treatment of serum samples
Day 1: Aliquot serum samples and start titration of the R3ΔPB1 virus
Day 2: Scan 384-well plates and calculate virus titer
Day 3: Start influenza neutralization assay
Day 4: Scan 384-well plates and calculate neutralization titers

C. Experimental procedures

C.1. RDE treatment

Animal and human sera contain various sialic acid containing glycans, which bind to the hemagglutinin (HA) of influenza virus and inhibit virus entry into the target cells. It is necessary to remove these nonspecific binders of viral hemagglutinin from serum samples before testing in the microneutralization assay by treating with receptor destroying enzyme (RDE). RDE is supplied as a filter-sterilized, lyophilized culture supernatant of Vibrio cholerae Ogawa type 558.

Materials and Supplies:

(a) Thawed samples stored on ice
(b) Receptor destroying enzyme RDE II (Accurate Chemical & Scientific Corp, Cat # YCC340-122).
(c) Physiological saline sterile solution 0.85% (Thermo Fisher, Cat # R064448)
(d) PBS pH 7.4, sterile (Quality Biological, Cat # 114-058-101)
(e) 2-ml Cryovials (Sarstedt, Fisher Scientific, Cat # 50-809-242)

Equipment:

(a) Biosafety cabinet (BSC)
(b) Water bath set at 37°C
(c) Water bath set at 56°C
(d) Freezer -20°C
(e) Refrigerator

Methods:
(a) Completely dissolve the lyophilized RDE II in 20 mL of sterile physiological saline (0.85%). This solution should be used immediately.
(b) Quickly thaw sera in 37°C water bath or in a heat block set at 37°C with water in each well for rapid heat transfer.
(c) Immediately after thawing, place sera in ice and keep on ice during use.
(d) Add 1 volume of serum sample to each tube.
(e) Add 3 volumes of RDE to each tube and mix.
(f) Incubate in water bath at 37°C for 18-20 hours.
(g) Heat inactivate the RDE/serum mixtures at 56°C for 30-60 min.
(h) May store at 4°C overnight. If longer storage is needed, aliquot and freeze at -20°C or colder.

C.2. Influenza neutralization assay (384-well) using R3ΔPB1 influenza viruses (Fluorescence detection with Celigo image-based plate reader)

The purpose of this protocol is to define the methods for the following experiments.

C.2.1. Titration of R3ΔPB1 influenza viruses using Celigo image-based plate reader
C.2.2. Neutralization assay using R3ΔPB1 influenza viruses

Materials and Supplies:
(a) 200 μl tips
(b) 20 μl tips
(c) 10- and 25-ml pipettes
(d) 15- and 50-ml conical tubes
(e) Reservoirs
(f) 384-well plate (Greiner Cat # 781091)
(g) 96-well U-bottom (sterile, not TC treated) (Falcon Cat # 351177)
(h) Trypsin, 0.25% 1X Solution, with 2.5 g Porcine Trypsin (1:250/L) in HBSS with 0.2 g/L EDTA, without Calcium or Magnesium (VWR Cat # 16777-166)
(i) PBS pH 7.4, sterile (Quality Biological Cat# 114-058-101)
(j) Opti-MEM (no Phenol RED, Thermo Fisher Cat # 11058021)
(k) Disposable 50-ml filter units (Corning, Cat # 430320)
(l) TPCK-treated trypsin (Sigma, Cat # T1426-50MG). 50 mg powder is mixed with 50 ml sterile water (Thermo Fisher, Cat # 4387937) at 1 mg/ml, solution is filtered with 50-ml disposable filter units and 200 µl aliquots are stored at -20°C for 1-2 years.

Equipment:
(a) Biosafety cabinet

(b) Microscope
(c) Portable Pipet-Aid
(d) Single channel pipettes (P2, P20, P200 and P1000)
(e) 8-channel pipette (20-200 µl)
(f) 12-channel pipette (20-200 µl)
(g) (optional) 24-well channel pipette (20-100 µl)
(h) (optional) Viaflo 96 Handheld Electronic 24,96 and 384 Channel Pipette (Integra)
(i) CO_2 incubator at 37ºC
(j) Refrigerator
(k) Water bath set at 37°C
(l) Centrifuge
(m) Plate reader (Celigo, Nexcelom; with customized red filter: EX 540/80 nm, DIC 593 nm and EM 593/LP nm)

Methods:

C.2.1. Titration of R3ΔPB1 influenza viruses using Celigo image-based plate reader

C.2.1.1. Prepare 384-well plates with PB1-expressing MDCK-SIAT1 cells
(a) Spray the surface of material and supplies with 70% ethanol and bring them into BSC.
(b) Warm up Opti-MEM medium at 37°C using the water bath.
(c) Take the flask with PB1-expressing MDCK-SIAT1 cells out from CO_2 incubator.
(d) Place the plate under the microscope.
(e) Observe the cells.
(f) Record the approximate cell confluence as a percent of surface area.
Note: Choose the wells with 40-60% cell confluency.
(g) Bring the plate into the BSC.
(h) Remove the supernatant gently without disturbing the cell monolayer.
(i) Wash cells twice with 20-30 ml PBS per T175 flaks.
(j) Remove PBS.
(k) Trypsinize with 0.25% Trypsin for 15-20 minutes.
(l) Tap the flaks every 5-10 min.
(m) Re-suspend cells in 5-10 ml PBS by pipetting up and down several times.
(n) Dilute to 30-50 ml PBS and spin at 1500 rpm for 5 min.
(o) Discard supernatant and wash twice with PBS (resuspend/spin/discard).
(p) Count cells and seed at 3,000 cells in 20 µl Opti-MEM (no phenol-red) per well.
(q) Return the plate to CO_2 incubator until virus dilutions are ready.

C.2.1.2. Prepare virus and sample dilutions
(a) Prepare 2-fold dilution of virus using Opti-MEM in 96-well U-bottom plate.
Note: Change tips after preparing each dilution.
(b) Add 80 µl Opti-MEM to each well.
(c) Add 80 µl virus stock to column 1, mix and transfer 80 µl to column 2, move by column using 8-channel pipette (see layout in **Appendix 1**).

(d) Prepare Opti-MEM with TPCK-treated trypsin (25 ml OPTI-MEM with 100 µl of 1mg/ml TPCK-treated trypsin, final concentration 4 µg/ml).
(e) Prepare a second 96-well plate with 60 µl of Opti-MEM-TPCK trypsin in each well. **Note:** Each well should have the same amount of TPCK-treated trypsin.
(f) Transfer 60 µl from the plate containing virus dilutions to the plate with Opti-MEM-TPCK. This will be a "mock" neutralization plate. **Note:** Use 24- or 12-channel pipette and use different tips for each virus. (optional) Incubate at 37^0C for 1 h.
(g) Bring the 384-well plate with PB1-expressing MDCK-SIAT1 cells from CO_2 incubator.
(h) Transfer 25 µl from 96-well "mock" neutralization plate to 384-well plate with cells. Use 24/12 channel pipette. Use different tips for each virus. Measure florescence at 18 h, 22 h, 24 h using Celigo.
(i) Plot total number of fluorescent cells against virus dilutions.
(j) The appropriate virus dilution to use in the microneutralization assay is the mid-point of the linear region of the sigmoidal curve. The number of fluorescent foci at this virus dilution should be between 500-1000 fluorescent foci per 384-well at 18 h post-infection.

C.2.2. Neutralization assay using R3 delPB1 influenza viruses

C.2.2.1. Prepare 384-well plates with PB1-expressing MDCK-SIAT1 cell as described in “C.2.1.1. Prepare 384-well plates with PB1-expressing MDCK-SIAT1 cell” in section “C.2.1. Titration of R3ΔPB1 influenza viruses”.

C.2.2.2. Prepare serum/antibody dilutions
(a) Bring Opti-MEM (no TPCK trypsin) to BSC.
(b) Prepare 4-fold dilutions of inactivated sera in 96-well U-bottom (sterile, not TC treated) (see layout in Appendix 2). Mix well, change tips after each dilution.
(c) Prepare 4-fold dilutions of control monoclonal antibodies (see layout in Appendix 3). Mix well, change tips after each dilution.
(d) (recommended) Use mAbs with known specificity as controls in each assay such as CR6261, CR8020 and MEDI8852.
(e) Transfer 20 µl solution from row to row starting with row A and ending at row H. After removing 20 µl from row H, discard. For each dilution mix 3-4 times by pipetting up and down without making bubbles and change the tips after each transfer.
(f) Thaw virus stock quickly by placing in the water bath at 37°C (store on ice after thawing if necessary).
(g) Dilute virus in Opti-MEM with TPCK-treated trypsin (final concentration 4 µg/ml TPCK-treated trypsin, stock concentration is 1 mg/ml). Save 0.5-1 ml solution of Opti-MEM with TPCK-treated trypsin for negative control wells (CC).
(h) Dilute pre-titrated virus as described in “C.2.1. Titration of R3ΔPB1 influenza viruses”.
(i) Add 60 µl of diluted virus to each of well of raw A to G.
(j) Row H has the controls (see layouts in **Appendixes 2** and **3**):
    i. Positive controls (Virus Control-VC): add 60 µl virus only to columns 1, 2, 3 and 7, 8, 9 (positive controls-VC: H1, H2, H3 and H7, H8, H9).

ii. Negative controls (Cell Control-CC): add 60 µl of Opti-MEM with TCPCK-treated trypsin to columns 4, 5, 6 and 10, 11, 12 (CC: H4, H5, H6 and H10, H11, H12).

Note: All wells should have the same amount of TPCK-treated trypsin.

(k) Incubate at 37ºC for 1 h.

(l) Transfer 25 µl of each neutralization reaction from 96-well to four wells of 384-well plate: from A1 of the 96-well plate to A1, A2, B1, and B2 of the 384-well plate (see layout in **Appendix 4**).

Note: We use 24-channel pipette (Rainin) or VIAFLO 96 (Integra).

Note: Mix well virus solution with cell suspension in 384-well. Change tips after each transfer.

(m) Scan the plate 18 h post infection using Celigo (optional: plates can be scanned again at 21 h and 24 h. We aim to have cca. 500-1000 fluorescent foci at 18 h post-infection.

Note: if numbers are lower than 500 fluorescent foci/well, plate should be read every 2-3 h up to 24 h post-infection until the number of fluorescent foci reaches 500-1000 per well.

(n) Calculate IC_{50} or IC_{80} for each sample.

Note: The percent neutralization was calculated by constraining the VC control as 0% and the CC control as 100% and plotted against antibody concentration. A curve fit was generated by a four-parameter nonlinear fit model in Prism.

Appendix 1: Layout of 96-well plate for virus titration

	1	2	3	4	5	6	7	8	9	10	11	12
A	virus:2	virus:4	virus:8	virus:16	virus:32	virus:64	virus:128	virus:256	virus:512	virus:1024	virus:2048	no virus
B	virus:2	virus:4	virus:8	virus:16	virus:32	virus:64	virus:128	virus:256	virus:512	virus:1024	virus:2048	no virus
C	virus:2	virus:4	virus:8	virus:16	virus:32	virus:32	virus:128	virus:256	virus:512	virus:512	virus:2048	no virus
D	virus:2	virus:4	virus:8	virus:16	virus:32	virus:32	virus:128	virus:256	virus:512	virus:512	virus:2048	no virus
E	virus:2	virus:4	virus:8	virus:16	virus:32	virus:32	virus:128	virus:256	virus:512	virus:512	virus:2048	no virus
F	virus:2	virus:4	virus:8	virus:16	virus:32	virus:32	virus:128	virus:256	virus:512	virus:512	virus:2048	no virus
G	virus:2	virus:4	virus:8	virus:16	virus:32	virus:32	virus:128	virus:256	virus:512	virus:512	virus:2048	no virus
H	virus:2	virus:4	virus:8	virus:16	virus:32	virus:32	virus:128	virus:256	virus:512	virus:512	virus:2048	no virus

Appendix 2: Layout of 96-well plate with serum dilutions

	1	2	3	4	5	6	7	8	9	10	11	12	
A	S1 Dil1	S2 Dil1	S3 Dil1	S4 Dil1	S5 Dil1	S6 Dil1	S7 Dil1	S8 Dil1	S9 Dil1	S10 Dil1	S11 Dil1	S12 Dil1	64 µl diluent + 16 µl of inactivated serum/well
B	S1 Dil2	S2 Dil2	S3 Dil2	S4 Dil2	S5 Dil2	S6 Dil2	S7 Dil2	S8 Dil2	S9 Dil2	S10 Dil2	S11 Dil2	S12 Dil2	60 µl diluent/well
C	S1 Dil3	S2 Dil3	S3 Dil3	S4 Dil3	S5 Dil3	S6 Dil3	S7 Dil3	S8 Dil3	S9 Dil3	S10 Dil3	S11 Dil3	S12 Dil3	60 µl diluent/well
D	S1 Dil4	S2 Dil4	S3 Dil4	S4 Dil4	S5 Dil4	S6 Dil4	S7 Dil4	S8 Dil4	S9 Dil4	S10 Dil4	S11 Dil4	S12 Dil4	60 µl diluent/well
E	S1 Dil5	S2 Dil5	S3 Dil5	S4 Dil5	S5 Dil5	S6 Dil5	S7 Dil5	S8 Dil5	S9 Dil5	S10 Dil5	S11 Dil5	S12 Dil5	60 µl diluent/well
F	S1 Dil6	S2 Dil6	S3 Dil6	S4 Dil6	S5 Dil6	S6 Dil6	S7 Dil6	S8 Dil6	S9 Dil6	S10 Dil6	S11 Dil6	S12 Dil6	60 µl diluent/well
G	S1 Dil7	S2 Dil7	S3 Dil7	S4 Dil7	S5 Dil7	S6 Dil7	S7 Dil7	S8 Dil7	S9 Dil7	S10 Dil7	S11 Dil7	S12 Dil7	60 µl diluent/well
H	VC	VC	VC	CC	CC	CC	VC	VC	VC	CC	CC	CC	60 µl diluent/well (add additional 60 µl diluent to CC as negative controls)

Appendix 3: Layout of 96-well plate with antibody dilutions

	1	2	3	4	5	6	7	8	9	10	11	12	
A	A1 Dil1	A2 Dil1	A3 Dil1	A4 Dil1	A5 Dil1	A6 Dil1	A7 Dil1	A8 Dil1	A9 Dil1	A10 Dil1	A11 Dil1	A12 Dil1	76 µl diluent + 4 µl of 1 mg/ml of mAb/well
B	A1 Dil2	A2 Dil2	A3 Dil2	A4 Dil2	A5 Dil2	A6 Dil2	A7 Dil2	A8 Dil2	A9 Dil2	A10 Dil2	A11 Dil2	A12 Dil2	60 µl diluent/well
C	A1 Dil3	A2 Dil3	A3 Dil3	A4 Dil3	A5 Dil3	A6 Dil3	A7 Dil3	A8 Dil3	A9 Dil3	A10 Dil3	A11 Dil3	A12 Dil3	60 µl diluent/well
D	A1 Dil4	A2 Dil4	A3 Dil4	A4 Dil4	A5 Dil4	A6 Dil4	A7 Dil4	A8 Dil4	A9 Dil4	A10 Dil4	A11 Dil4	A12 Dil4	60 µl diluent/well
E	A1 Dil5	A2 Dil5	A3 Dil5	A4 Dil5	A5 Dil5	A6 Dil5	A7 Dil5	A8 Dil5	A9 Dil5	A10 Dil5	A11 Dil5	A12 Dil5	60 µl diluent/well
F	A1 Dil6	A2 Dil6	A3 Dil6	A4 Dil6	A5 Dil6	A6 Dil6	A7 Dil6	A8 Dil6	A9 Dil6	A10 Dil6	A11 Dil6	A12 Dil6	60 µl diluent/well
G	A1 Dil7	A2 Dil7	A3 Dil7	A4 Dil7	A5 Dil7	A6 Dil7	A7 Dil7	A8 Dil7	A9 Dil7	A10 Dil7	A11 Dil7	A12 Dil7	60 µl diluent/well
H	VC	VC	VC	CC	CC	CC	VC	VC	VC	CC	CC	CC	60 µl diluent/well (add additional 60 µl diluent to CC as negative controls)

Appendix 4: Layout of influenza neutralization plate 384-well plate

	1	2	3	4	5	6	7	8	9	10	11	12	13	14	15	16	17	18	19	20	21	22	23	24
A	1	1	1	1	1	1	1	1	1	1	1	1	1	1	1	1	1	1	1	1	1	1	1	1
B	1	1	1	1	1	1	1	1	1	1	1	1	1	1	1	1	1	1	1	1	1	1	1	1
C	2	2	2	2	2	2	2	2	2	2	2	2	2	2	2	2	2	2	2	2	2	2	2	2
D	2	2	2	2	2	2	2	2	2	2	2	2	2	2	2	2	2	2	2	2	2	2	2	2
E	3	3	3	3	3	3	3	3	3	3	3	3	3	3	3	3	3	3	3	3	3	3	3	3
F	3	3	3	3	3	3	3	3	3	3	3	3	3	3	3	3	3	3	3	3	3	3	3	3
G	4	4	4	4	4	4	4	4	4	4	4	4	4	4	4	4	4	4	4	4	4	4	4	4
H	4	4	4	4	4	4	4	4	4	4	4	4	4	4	4	4	4	4	4	4	4	4	4	4
I	5	5	5	5	5	5	5	5	5	5	5	5	5	5	5	5	5	5	5	5	5	5	5	5
J	5	5	5	5	5	5	5	5	5	5	5	5	5	5	5	5	5	5	5	5	5	5	5	5
K	6	6	6	6	6	6	6	6	6	6	6	6	6	6	6	6	6	6	6	6	6	6	6	6
L	6	6	6	6	6	6	6	6	6	6	6	6	6	6	6	6	6	6	6	6	6	6	6	6
M	7	7	7	7	7	7	7	7	7	7	7	7	7	7	7	7	7	7	7	7	7	7	7	7
N	7	7	7	7	7	7	7	7	7	7	7	7	7	7	7	7	7	7	7	7	7	7	7	7
O	VC	VC	VC	VC	VC	VC	CC	CC	CC	CC	CC	CC	VC	VC	VC	VC	VC	VC	CC	CC	CC	CC	CC	CC
P	VC	VC	VC	VC	VC	VC	CC	CC	CC	CC	CC	CC	VC	VC	VC	VC	VC	VC	CC	CC	CC	CC	CC	CC

Supplementary Methods

Quantitative RT-PCR

Viral RNA was extracted from cell culture supernatants with Qiamp Viral RNA mini kit (Qiagen). cDNA and qPCR was prepared using the Taqman Fast Virus 1-step. qRT-PCRT primers for influenza H1, H3 HAs and M genes are described below. qRT-PCR was performed using real-time Biorad CFX96. The number of genome copies was inferred from standard curves prepared for each gene with viral RNA of A/Puerto Rico/8/1934 (ATCC: VR-95PQ) and A/Hong Kong/8/1963 (H3N2) (ATCC: VR-544PQ), that have the genome copy number determined by droplet digital PCR.

Human samples

All human serum samples for this study were collected with informed consent of volunteers, and approval for this study was obtained under protocol number VRC 310 and VRC 315 (Clinicaltrials.gov NCT01086657 and NCT02206464, respectively). VRC 310 is a single-site, phase 1, open-label, randomized clinical trial to evaluate the safety, tolerability, and immunogenicity of prime-boost vaccination regimens against H5N1 influenza conducted at the National Institutes of Health (NIH) Clinical Center by the VRC, NIAID (Clinicaltrial.gov NCT01086657)[1]. VRC 315 is a single-site, phase 1, open-label, randomized clinical trial to evaluate the safety, tolerability, and immunogenicity of prime-boost vaccination regimens against H7N9 Influenza conducted at the NIH Clinical Center by the VRC, NIAID (Clinicaltrial.gov NCT02206464)[2]. These studies were approved by the NIAID Intramural Institutional Review Board. US Department of Health and Human Services guidelines for conducting clinical research were followed. Primary and secondary outcome measures of NCT01086657 were adverse events, including clinical, laboratory and local and systemic reactogenicity; and immunogenicity as measured by humoral and cellular assays, respectively. Primary and secondary outcome measures of NCT02206464 were solicited adverse events (reactogenicity), adverse events of all severities, serious adverse events, new chronic medical conditions and influenza-like illnesses; and H7-specific antibody response as measured by HAI assay, respectively. Samples were collected between February 2010 and December 2011 (NCT01086657) or between July 2014 and January 2016 (NCT02206464).

Neutralization assay of human polyclonal sera

Serum samples used in the assays were collected before and after experimental vaccine administrations (2 weeks post second immunization). Four-fold serial dilutions of human sera pretreated with receptor destroying enzyme II (RDE) (Denka Seiken, Japan) and heat inactivated at 56°C for 40 minutes were mixed 1:1 with pre-titrated replication-restricted reporter viruses for 1 hour at 37°C. The mixture was then added to cells in quadruplicate in a 384-well plate containing 10,000 MDCK-SIAT1-PB1 cells in each well. After overnight incubation at 37°C, the number of fluorescent cells in each well was counted automatically using a Celigo image cytometer (Nexcelom Biosciences) using Target 1 protocol as implemented in the Celigo software. The percent neutralization was calculated by constraining the virus control as 0% and the cell-only control at 100% and plotting against serum concentration. A curve fit was generated by a four-parameter nonlinear fit model in Prism (GraphPad). The 80% inhibitory (IC_{80}) concentrations were obtained from the curve fit for each serum sample.

Fluorescent plaque reduction assay

96-well black plates with transparent bottom were seeded with 50,000 cells well^{-1} MDCK-SIAT PB1 cells. Next day, cells were washed intensively with PBS. Four-fold serial dilutions of monoclonal antibodies 1G01 (ref. 3), CD6 (ref. 4), MEDI8852 (ref. 5) and anti-RSV F D25 (ref. 6) were prepared in triplicate in a low protein binding 96-well plate (Corning) in 1 $\times$ MEM (Gibco). 50 µl of each dilution was added to the cells. The starting concentration for anti-NA, 1G01 and CD6, and anti-RSV F monoclonal antibodies was 100 µg ml^{-1} and for anti-HA monoclonal antibody, MEDI8852, was 25 µg ml^{-1}. Next, 50 µl of pre-titrated R3ΔPB1 A/California/07/2009 H1N1 in MEM was added to the cells, mixed with the monoclonal antibody and incubated for 1 h at 37°C. Control wells of virus alone (VC) and diluent alone (CC) were included on each plate. After 1 h incubation, 3% Avicel solution was mixed in equal volumes with 2 $\times$ MEM. One hundred µl of this Avicel mixture was added to each well, gently mixed with the virus-antibody mixture and incubated for at 37°C. TPCK-treated trypsin (Sigma) was added at 1 µg ml^{-1} final concentration. Area of fluorescent cells in each well was counted automatically without removing the overlay using Confluence 1 protocol as implemented in Celigo image cytometer (Nexcelom Biosciences) at 24, 32 and 48 h post infection. The percent inhibition was calculated as described above. The IC_{80} concentrations were obtained from the curve fit for each antibody.

Supplementary References

1. Ledgerwood, J. E. *et al.* Prime-boost interval matters: a randomized phase 1 study to identify the minimum interval necessary to observe the H5 DNA influenza vaccine priming effect. *J Infect Dis* **208**, 418-422, doi:10.1093/infdis/jit180 (2013).
2. DeZure, A. D. *et al.* An avian influenza H7 DNA priming vaccine is safe and immunogenic in a randomized phase I clinical trial. *NPJ Vaccines* **2**, 15, doi:10.1038/s41541-017-0016-6 (2017).
3. Stadlbauer, D. *et al.* Broadly protective human antibodies that target the active site of influenza virus neuraminidase. *Science* **366**, 499-504, doi:10.1126/science.aay0678 (2019).
4. Wan, H. *et al.* Structural characterization of a protective epitope spanning A(H1N1)pdm09 influenza virus neuraminidase monomers. *Nat Commun* **6**, 6114, doi:10.1038/ncomms7114 (2015).
5. Kallewaard, N. L. *et al.* Structure and Function Analysis of an Antibody Recognizing All Influenza A Subtypes. *Cell* **166**, 596-608, doi:10.1016/j.cell.2016.05.073 (2016).
6. McLellan, J. S. *et al.* Structure-based design of a fusion glycoprotein vaccine for respiratory syncytial virus. *Science* **342**, 592-598, doi:10.1126/science.1243283 (2013).
