# Supplementary material for: A comprehensive influenza reporter virus panel for high-throughput deep profiling of neutralizing antibodies
Source: Nat Commun. 2021 Mar 19;12:1722. doi: 10.1038/s41467-021-21954-2 (PMC7979723; doi:10.1038/s41467-021-21954-2)
Supplement: Supplementary file 2 — Reporting Summary [file 41467_2021_21954_MOESM2_ESM.pdf]

Corresponding author(s): Barney Graham and Masaru Kanekiyo

Last updated by author(s): Feb 12, 2021

Reporting Summary

Nature Research wishes to improve the reproducibility of the work that we publish. This form provides structure for consistency and transparency in reporting. For further information on Nature Research policies, see our Editorial Policies and the Editorial Policy Checklist.

Statistics

For all statistical analyses, confirm that the following items are present in the figure legend, table legend, main text, or Methods section.

n/a	Confirmed	
☐	☒	The exact sample size (*n*) for each experimental group/condition, given as a discrete number and unit of measurement
☐	☒	A statement on whether measurements were taken from distinct samples or whether the same sample was measured repeatedly
☐	☒	The statistical test(s) used AND whether they are one- or two-sided<br>*Only common tests should be described solely by name; describe more complex techniques in the Methods section.*
☒	☐	A description of all covariates tested
☒	☐	A description of any assumptions or corrections, such as tests of normality and adjustment for multiple comparisons
☐	☒	A full description of the statistical parameters including central tendency (e.g. means) or other basic estimates (e.g. regression coefficient) AND variation (e.g. standard deviation) or associated estimates of uncertainty (e.g. confidence intervals)
☐	☒	For null hypothesis testing, the test statistic (e.g. *F*, *t*, *r*) with confidence intervals, effect sizes, degrees of freedom and *P* value noted<br>*Give P values as exact values whenever suitable.*
☒	☐	For Bayesian analysis, information on the choice of priors and Markov chain Monte Carlo settings
☒	☐	For hierarchical and complex designs, identification of the appropriate level for tests and full reporting of outcomes
☐	☒	Estimates of effect sizes (e.g. Cohen's *d*, Pearson's *r*), indicating how they were calculated

Our web collection on statistics for biologists contains articles on many of the points above.

Software and code

Policy information about availability of computer code

Data collection	Plate image acquisition: Celigo v4.1
Data analysis	All data with the following exceptions were analyzed with GraphPad Prism 8.<br>Phylogenetic analysis: MEGA v10<br>Sequence alignment (large sets, more than one thousand sequences): MAFFT (online version) v7<br>Sequence alignment (less than one thousand sequences): Muscle Wrapper as implemented in BioEdit 7.2.5, Sequencher 5.4.6<br>Sequence analysis: CD-HIT EST (http://weizhong-lab.ucsd.edu/cdhit-web-server/cgi-bin/index.cgi?cmd=cd-hit-est)<br>Structural conservation: ConSurf server (https://consurf.tau.ac.il/)<br>Structure rendering: UCSF Chimera 1.13.1<br>Heatmap analysis: ClustVis server (https://biit.cs.ut.ee/clustvis/)<br>Graphing: Graphpad Prism v8<br>Figure compilation: Inkscape 1.0.0<br>Tissue culture infectious dose calculation: Reed–Muench method<br>Sequence curation: GISAID database (http://platform.gisaid.org)

For manuscripts utilizing custom algorithms or software that are central to the research but not yet described in published literature, software must be made available to editors and reviewers. We strongly encourage code deposition in a community repository (e.g. GitHub). See the Nature Research guidelines for submitting code & software for further information.

Data

Policy information about availability of data

All manuscripts must include a data availability statement. This statement should provide the following information, where applicable:

- Accession codes, unique identifiers, or web links for publicly available datasets
- A list of figures that have associated raw data
- A description of any restrictions on data availability

All images and data were generated and analyzed by the authors, and will be made available by the corresponding authors (B.S.G. and M.K.) upon reasonable request. Influenza reverse genetics plasmids were obtained from St. Jude Research Hospital thorough an MTA. All sequences corresponding to the influenza HA, NA and reporter segments used in the present study have been deposited to NCBI Genbank under accession numbers MW298159–MW298274. Source data corresponding to Figs. 2–6 and Supplementary figs. 2–7 are provided as Source Data.

Field-specific reporting

Please select the one below that is the best fit for your research. If you are not sure, read the appropriate sections before making your selection.

- [x] Life sciences
- [ ] Behavioural & social sciences
- [ ] Ecological, evolutionary & environmental sciences

For a reference copy of the document with all sections, see nature.com/documents/nr-reporting-summary-flat.pdf

Life sciences study design

All studies must disclose on these points even when the disclosure is negative.

Sample size	No sample-size calculation was performed. For the experiments, sample sizes were determined based on our previous studies utilizing similar experimental techniques.
Data exclusions	No data has been excluded.
Replication	All analyses for antibody binding, specificity, and virus neutralization assays have been performed at least twice or more with similar results.
Randomization	No randomization was performed. Human samples were obtained from our previously conducted open-label phase I trials.
Blinding	Experimenters were blinded to experimental conditions whenever possible. Readout of the serological assays including virus neutralization assays, and structural, biochemical and biophysical characterizations were not performed in a blinded manner, as these experiments often require subtle real-time adjustment to ensure optimal data collection.

Reporting for specific materials, systems and methods

We require information from authors about some types of materials, experimental systems and methods used in many studies. Here, indicate whether each material, system or method listed is relevant to your study. If you are not sure if a list item applies to your research, read the appropriate section before selecting a response.

Materials & experimental systems

n/a	Involved in the study	
[ ]	[x]	Antibodies
[ ]	[x]	Eukaryotic cell lines
[x]	[ ]	Palaeontology and archaeology
[x]	[ ]	Animals and other organisms
[ ]	[x]	Human research participants
[ ]	[x]	Clinical data
[x]	[ ]	Dual use research of concern

Methods

n/a	Involved in the study	
[x]	[ ]	ChIP-seq
[x]	[ ]	Flow cytometry
[x]	[ ]	MRI-based neuroimaging

Antibodies

Antibodies used	All the antibodies used in the study were made recombinantly by cloning antibody heavy and light chains into the respective mammalian expression vectors. Antibodies were produced in mammalian cells (Expi293 cells) by transient transfection of expression vectors and purified by protein A affinity chromatography. Sequences, specificity and function of the antibodies were verified for each antibody.<br>Antibodies used in this study:<br>315-02-1F07; 315-09-1B12; 315-27-1C08; 315-53-1A09; 315-53-1B06; and 315-53-1F12 (Andrews, et al., Sci Immunol. 2017)<br>315-02-1H01 (Corbett, et al., mBio 2019)<br>315-19-1D12; 315-23-1C09; 315-53-1A07; 315-55-1E08; and 315-55-1E11 (this study)

	CR6261 (Throsby, et al., Plos One 2008)<br>CR8020 (Ekiert, et al., Science 2011)<br>CR9114 (Dreyfus, et al., Science 2012)<br>MEDI8852 (Kallewaard, et al., Cell 2016)<br>CT149 (Wu, et al., Nat Commun. 2015)<br>FI6v3 (Corti, et al., Science 2011)<br>CH65 (Whittle, et al., PNAS 2011)<br>5J8 (Krause, et al., J Virol. 2011)<br>C05 (Ekiert, et al., Nature 2012)<br>F045-092 (Ohshima, et al., J Virol. 2011)<br>F005-126 (Iba, et al., J Virol. 2014)<br>310-33-1F04 (Kanekiyo, et al., Nat Immunol. 2019)<br>Biotin-conjugated anti-influenza nucleoprotein antibodies (Millipore Sigma, Clone A1 and Clone A3, Catalog MAB8257B and MAB8258B, respectively)
Validation	All the antibodies used in the study were tested for their reactivity and specificity by ELISA, BLI using a set of recombinant HAs, or virus neutralization assays with multiple subtype viruses prior to use in the study.

Eukaryotic cell lines

Policy information about cell lines

Cell line source(s)	Expi293F (ThermoFisher, Catalog A14527); Flp-In 293 (Thermo-Fisher, Catalog R75007); HEK-293-PB1 (a gift from Dr. Jesse Bloom, Fred Hutchinson Cancer Research Center); MDCK-SIAT1 (Millipore Sigma, Catalog 05071502); MDCK-SIAT1-PB1 (this study); MDCK-SIAT1-HA (this study); Flp-In 293-HA (this study)
Authentication	Commercial cell lines were authenticated by manufacturers and no further authentications were performed by the authors; MDCK-SIAT-PB1, MDCK-SIAT1-HA, HEK-293-PB1, and Flp-In 293-HA cells were not authenticated. All cells used in the studies were not extensively passaged.
Mycoplasma contamination	Tested negative.
Commonly misidentified lines<br>(See ICLAC register)	No commonly misidentified cell lines were used in this study.

Human research participants

Policy information about studies involving human research participants

Population characteristics	Healthy adults aged 18–60 years with no history of H5 influenza vaccination were eligible, both sexes (NCT01086657), or healthy adults aged 18–60 years with no previous H7 avian influenza investigational vaccine administration (NCT02206464)
Recruitment	NCT01086657 (VRC 310) is a single-site, phase 1, open-label, randomized clinical trial conducted at the National Institutes of Health (NIH) Clinical Center by the NIAID VRC (Ledgerwood, et al., J Infect Dis. 2013). NCT02206464 (VRC 315) is a single-site, phase 1, open-label, randomized clinical trial conducted at the National Institutes of Health (NIH) Clinical Center by the NIAID VRC (DeZure, et al., NPJ Vaccines. 2017). These studies were approved by the NIAID Intramural Institutional Review Board. A total of 64 healthy adult subjects were enrolled between 8 March and 13 May 2010 (NCT01086657). The study included 34 men and 30 women, with a mean age (± s.d.) of 34 ± 11 years and a mean body mass index (± s.d.) of 26.8 ± 5.4 (NCT01086657). A total of 30 healthy individuals were enrolled between January 20, 2015 and March 11, 2015 (NCT02206464). Subject inclusion criteria also included a body-mass index lower than 40, normal baseline blood counts, and normal liver and renal function laboratory measurements (NCT02206464).
Ethics oversight	Intramural Institutional Review Board (IRB) of the National Institute of Allergy and Infectious Diseases, National Institutes of Health approved all the human studies. U.S. Department of Health and Human Services guidelines for conducting clinical research were followed.

Note that full information on the approval of the study protocol must also be provided in the manuscript.

Clinical data

Policy information about clinical studies

All manuscripts should comply with the ICMJEguidelines for publication of clinical research and a completedCONSORT checklist must be included with all submissions.

Clinical trial registration	NCT01086657 (VRC 310) and NCT02206464 (VRC 315)
Study protocol	VRC 310 (https://clinicaltrials.gov/ct2/show/NCT01086657?term=NCT01086657&rank=1); VRC 315 (https://clinicaltrials.gov/ct2/show/NCT02206464?term=NCT02206464&draw=2&rank=1)
Data collection	VRC 310 (February 2010–December 2011; Location, National Institutes of Health Clinical Center, Bethesda, MD); VRC 315 (July 2014–January 2016; Location, National Institutes of Health Clinical Center, Bethesda, MD)
Outcomes	VRC 310 (Primary Outcome Measures, adverse events, including clinical, laboratory and local and systemic reactogenicity; Secondary Outcome Measures, immunogenicity as measured by humoral and cellular assays); VRC 315 (Primary Outcome Measures, solicited

adverse events (reactogenicity), adverse events of all severities, serious adverse events, new chronic medical conditions and influenza-like illnesses; Secondary Outcome Measures, H7-specific antibody response as measured by HAI assay)
